# Supplementary material for: Group 3 medulloblastoma transcriptional networks collapse under domain specific EP300/CBP inhibition
Source: Nat Commun. 2024 Apr 25;15:3483. doi: 10.1038/s41467-024-47102-0 (PMC11045757; doi:10.1038/s41467-024-47102-0)
Supplement: Supplementary file 1 — Supplementary Information [file 41467_2024_47102_MOESM1_ESM.pdf]

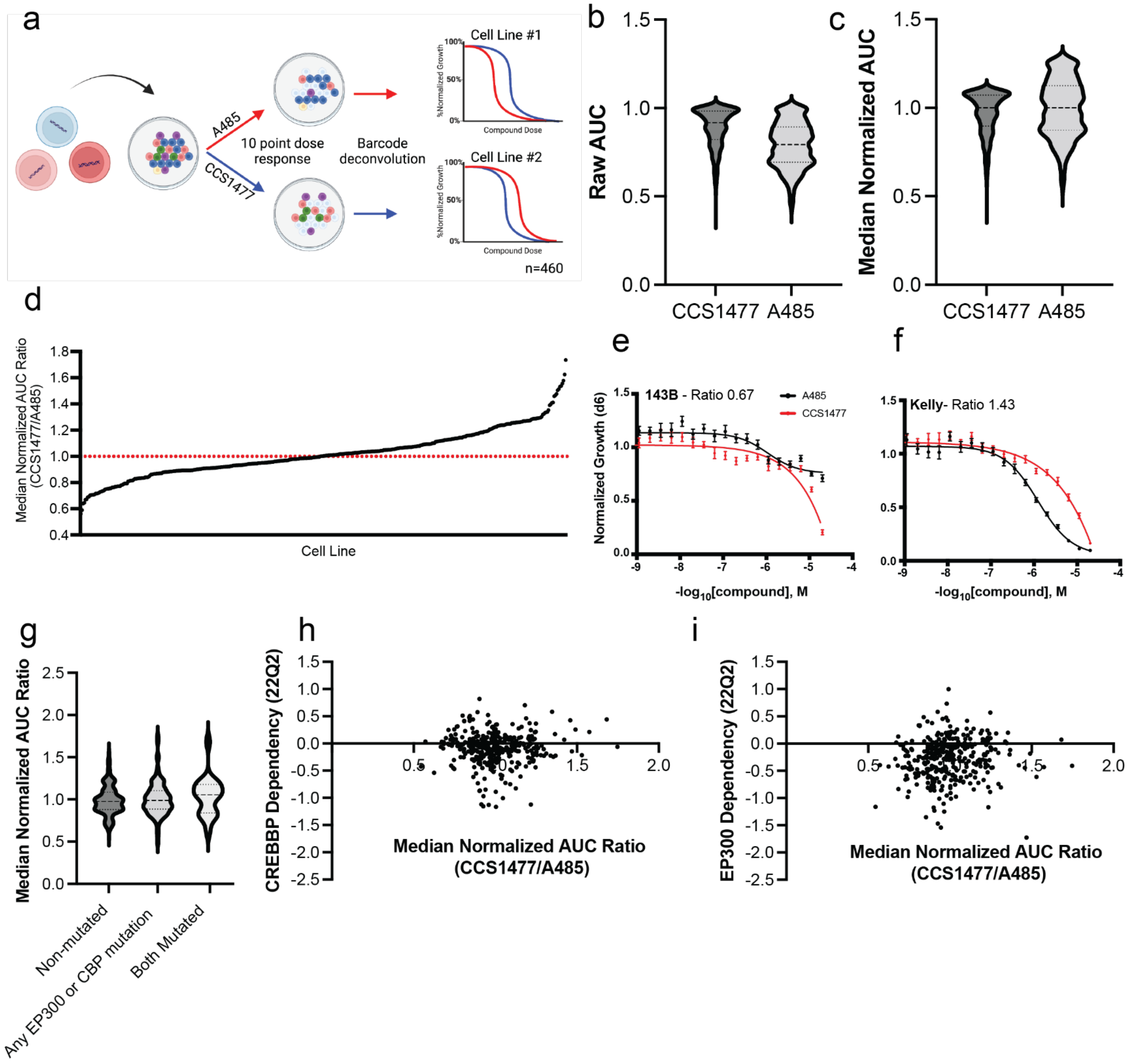

Supplementary Figure 1. PRISM screening validation for CCS1477 and A485 across cancer cell lines. A. Schematic of PRISM screen. Figure made in Biorender.com. **B.** Raw plotted AUC values of all cell lines (n = 460) treated with either CCS1477 or A485 for 5 days, resolved by PRISM barcode sequencing. Median shown by dashed central line, S.D. dotted lines. **C.** Median normalized AUCs of all cell lines (n = 460) treated with either CCS1477 or A485 for 5 days, resolved by PRISM barcode sequencing. Median shown by dashed central line, S.D. dotted lines, **D.** Individual cell lines plotted by median normalized AUC ratio (CCS1477/A485). Data from **A-C** is found in **Supplementary Tables 1,2**. **E,F.** 143B (E) and Kelly (F) cells were tested for dose-response effects of CCS1477 and A485 after six days by Cell-Titer Glo assay. n = 3 independent biological replicates for each dose. Error bars represent S.E.M. Ratio = median normalized AUC ratio. **G.** CCLE mutational data was extracted and compared with median normalized AUC ratio. The relationship of median normalized AUC ratio to cell line mutational status of *EP300*, *CBP* was determined. N = 328 (non-mutated), 97 (either *EP300* or *CBP* mutated), 30 (both *EP300* and *CBP* mutated), p=NS. **H,I.** Genetic dependency on *CBP* **(H)** or *EP300* **(I)** demonstrated compared with median normalized AUC ratio. Data extracted from www.depmap.org, 22Q4 dataset.

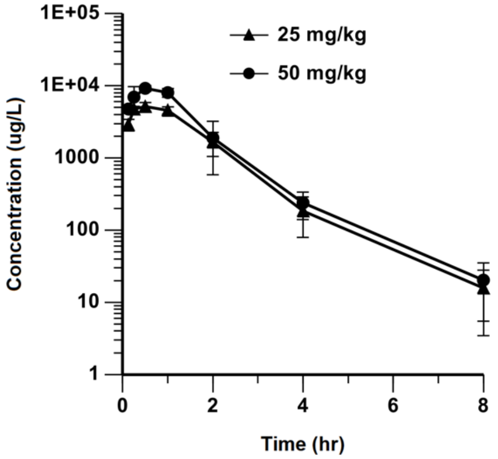

Parameter	Units	0.5 mg/kg IV	3 mg/kg PO	30 mg/kg PO	25 mg/kg IP (SJ)	50 mg/kg IP (SJ)
Cmax/C0	ng/mL	454	1010	9260	5150	9220
AUCinf	hr-ng/mL	595	3950	64600	8810	13600
T1/2	hr	0.96	1.71	3.59	0.862	0.941
CL(/F)	L/hr/kg	0.84	0.760	0.464	2.83	3.68
Vd(/F)	L/kg	1.1	1.86	2.41	3.52	5.00
Cmax/D	(ng/mL)/(mg/kg)	0.908	0.336	0.309	0.206	0.184
AUCinf/D	(hr-ng/mL)/(mg/kg)	1.19	1.32	2.15	0.353	0.272
F_est (PO,IP)	-	-	1.11	1.81	0.297	0.229

Supplementary Figure 2. Plasma pharmacokinetic analysis of CCS1477 following intraperitoneal injection in CD1 nude mice. Female CD1 nude mice were administered CCS1477 in 10% DMSO, 90% sterile water containing 20% hydroxy-propyl-cyclodextran by IP injection, and plasma recovered for analysis following retro-orbital puncture at timepoints following injection. n = 3 mice per timepoint, shown as mean and S.D. IV and oral (PO) dosing data retrieved from[1] for comparison. Plasma and brain CCS1477 concentrations were all below their respective lower limits of quantitation (1 and 6 ng/mL) at 16 and 24 hrs, and thus not displayed. Abbreviations: Cmax, maximum observed plasma concentration; C0, back-extrapolated concentration at time zero for an IV dose; AUCinf, area under the concentration-time curve extrapolated to infinity; T1/2, terminal half life; CL(/F), total systemic plasma clearance (or apparent extravascular plasma clearance); Vd(/F), volume of distribution (or apparent extravascular volume of distribution); Cmax/D, dose normalized Cmax or C0; AUCinf/D, dose normalized AUCinf; F_est, estimated fractional bioavailability by the extravascular route.

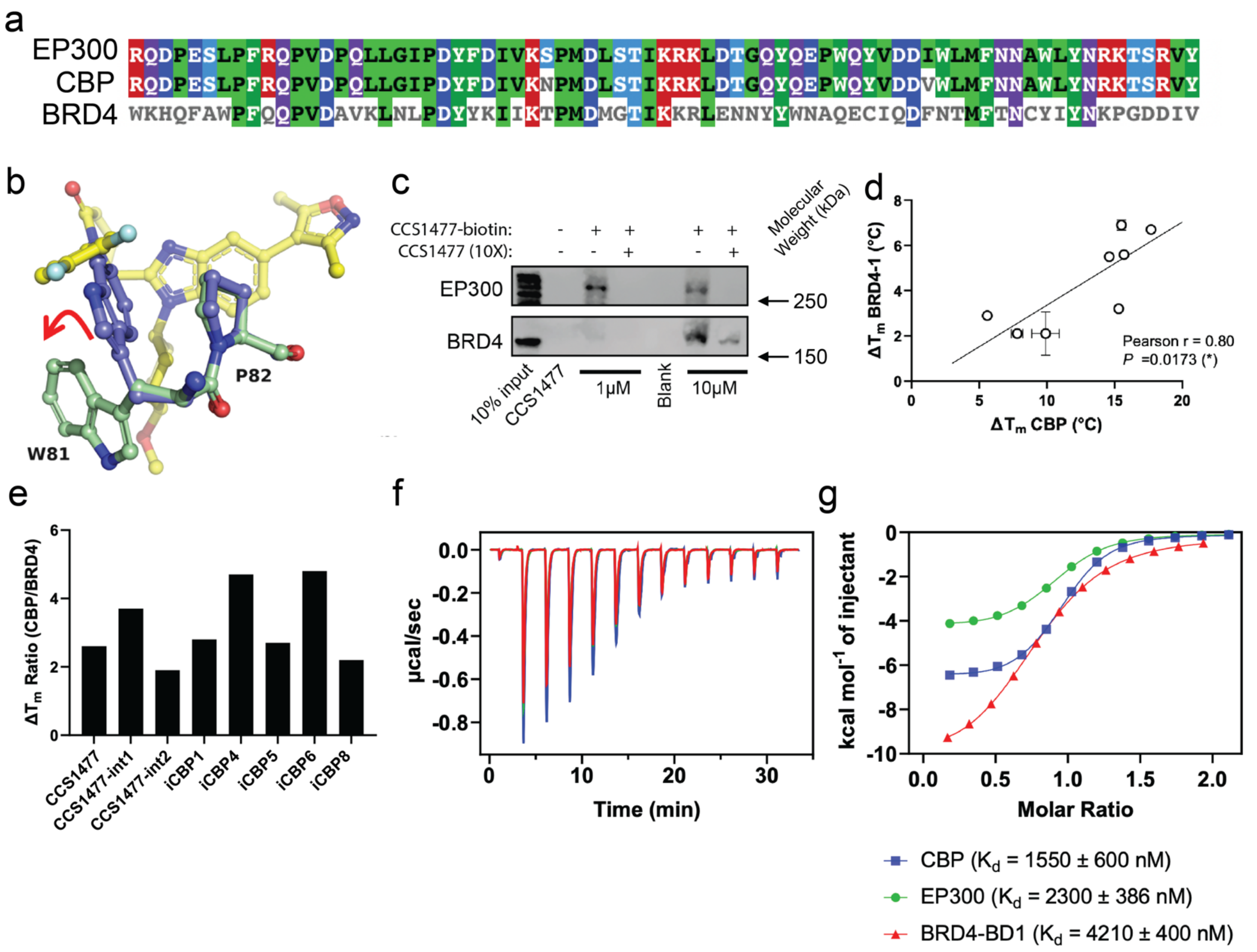

Supplementary Figure 3. Differential binding affinity of CCS1477 and analogues thereof for BRD4-BD1. A. Structural alignment of the bromodomains of EP300, CBP and BD1 of BRD4 demonstrate high amino acid sequence identity between EP300 and CBP, with lowered identity with BRD4. **B.** Overlay of the WPF shelf of BRD4-BD1 liganded with a fragment-like dimethylisoxazole (PDB 6FT3, blue) and CCS1477 (PDB 8FVK, green). W81 undergoes a conformational change as a result of steric hindrance upon binding of CCS1477. **C.** Biotinylated-CCS1477 pulldowns in Kelly neuroblastoma cell lysates demonstrates pulldown of EP300 but not BRD4 at low concentrations, and interaction with EP300 and BRD4 at higher concentrations of compound. Data is representative of n=3 independent lysates and reactions. **D.** Correlation of ΔT_m values determined by DSF for CBP and BRD4-BD1 interaction with the analogues of this study. Pearson's r and statistical significance p values (two-tailed) are indicated. The error bars represent the standard deviation of the DSF experiments, n = 3). **E.** Ratio of ΔTm values (CBP/BRD4) indicating changes in target specificity relative to CCS1477. **F,G.** ITC thermograms of the interaction of CCS1477int(1) (compound 1) with the bromodomains of CBP, EP300 and BRD4 (single experiment; error represents the S.E.M, from the data fit).

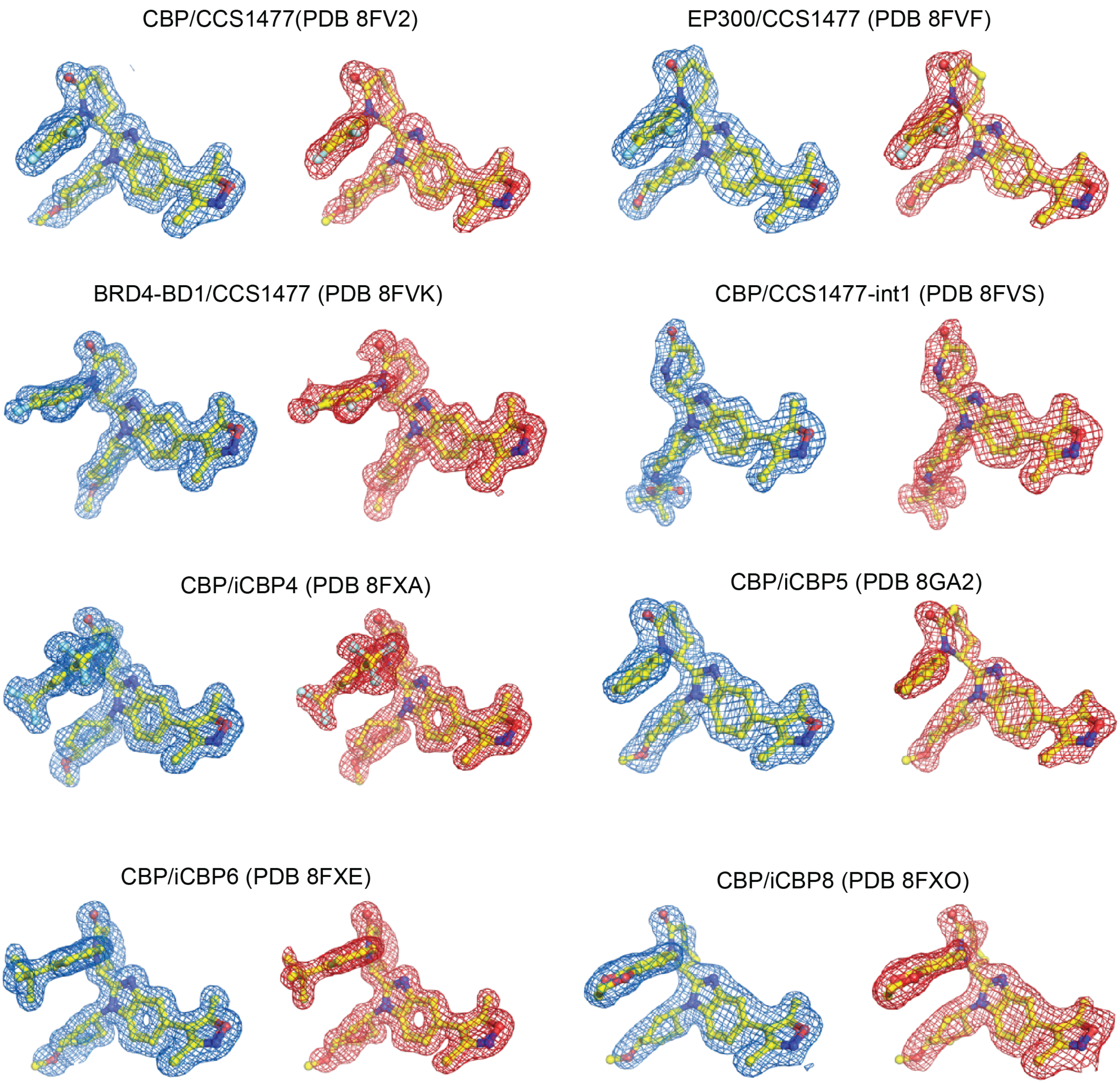

Supplementary Figure 4. Electron density maps of ligands bound to CBP, EP300 or BRD4-BD1. The left panel shows the 2Fo-Fc density map upon refinement with ligand (blue mesh, contoured at 1σ). The right panel shows the Fo-Fc density map upon refinement omitting the ligand (red mesh, contoured at 3σ).

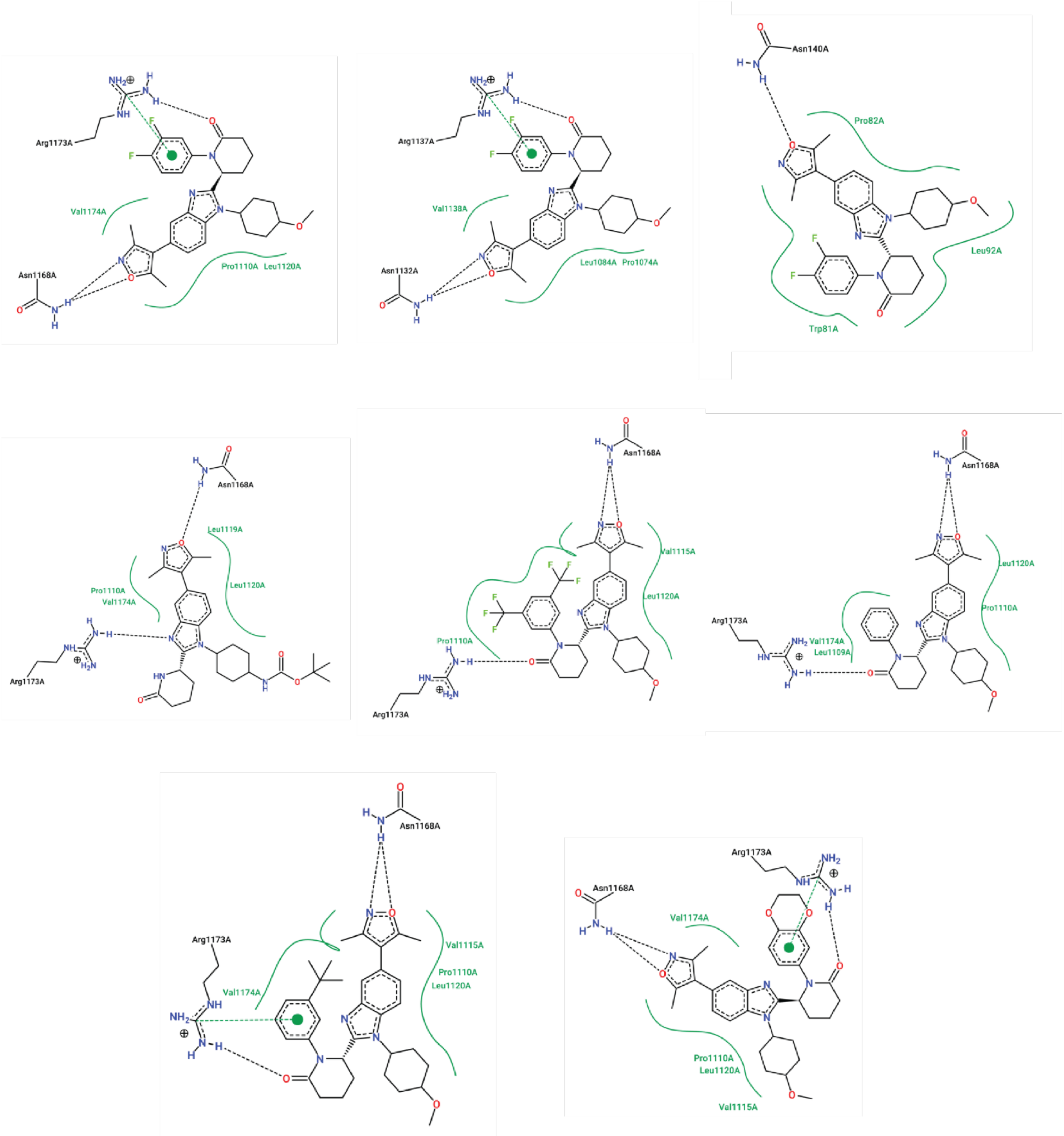

Supplementary Figure 5. 2D diagrams of the binding interactions of compounds in cocrystal structures with CBP, EP300 or BRD4 as computed by Poseview (https://proteins.plus/).

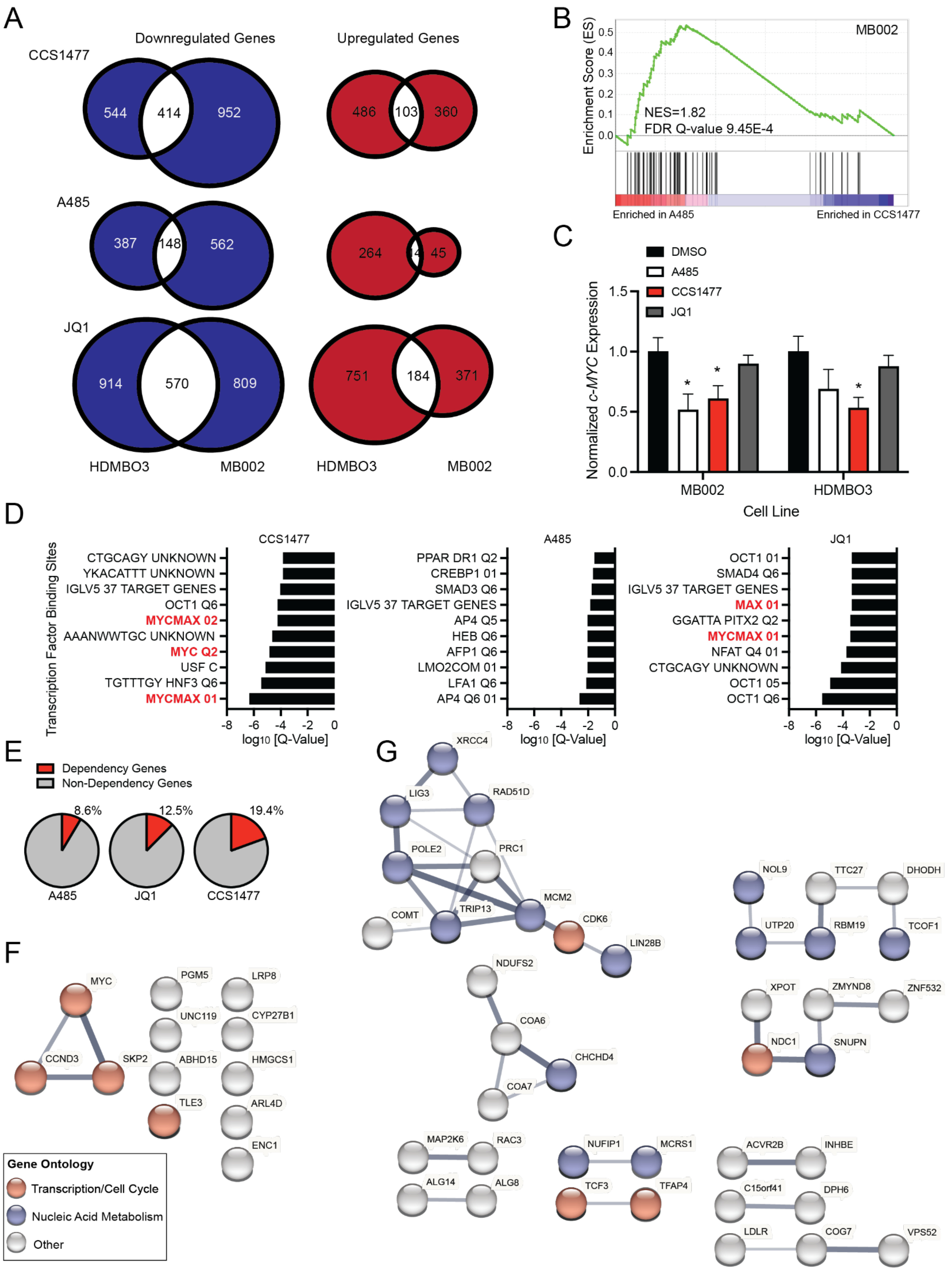
A
Downregulated Genes
Upregulated Genes
CCS1477
544
414
952
486
103
360
A485
387
148
562
264
14
45
JQ1
914
570
809
751
184
371
HDMBO3
MB002
HDMBO3
MB002
B
MB002
Enrichment Score (ES)
NES=1.82
FDR Q-value 9.45E-4
Enriched in A485
Enriched in CCS1477
C
DMSO
A485
CCS1477
JQ1
Normalized c-MYC Expression
MB002
HDMBO3
Cell Line
D
Transcription Factor Binding Sites
CCS1477
CTGCAGY UNKNOWN
YKACATTT UNKNOWN
IGLV5 37 TARGET GENES
OCT1 Q6
MYCMAX 02
AAANWWTGC UNKNOWN
MYC Q2
USF C
TGTTTGY HNF3 Q6
MYCMAX 01
log10 [Q-Value]
A485
PPAR DR1 Q2
CREBP1 01
SMAD3 Q6
IGLV5 37 TARGET GENES
AP4 Q5
HEB Q6
AFP1 Q6
LMO2COM 01
LFA1 Q6
AP4 Q6 01
log10 [Q-Value]
JQ1
OCT1 01
SMAD4 Q6
IGLV5 37 TARGET GENES
MAX 01
GGATTA PITX2 Q2
MYCMAX 01
NFAT Q4 01
CTGCAGY UNKNOWN
OCT1 05
OCT1 Q6
log10 [Q-Value]
E
Dependency Genes
Non-Dependency Genes
8.6%
12.5%
19.4%
A485
JQ1
CCS1477
F
MYC
CCND3
SKP2
TLE3
PGM5
UNC119
ABHD15
LRP8
CYP27B1
HMGCS1
ARL4D
ENC1
Gene Ontology
Transcription/Cell Cycle
Nucleic Acid Metabolism
Other
G
XRCC4
LIG3
RAD51D
POLE2
PRC1
MCM2
COMT
TRIP13
CDK6
LIN28B
NDUFS2
COA6
CHCHD4
COA7
NOL9
TTC27
DHODH
UTP20
RBM19
TCOF1
XPOT
ZMYND8
ZNF532
NDC1
SNUPN
MAP2K6
RAC3
ALG14
ALG8
NUFIP1
MCRS1
TCF3
TFAP4
ACVR2B
INHBE
C15orf41
DPH6
LDLR
COG7
VPS52

Supplementary Fig 6. CCS1477, but not A485 or JQ1, disrupts a densely interacting network of genes implicated in transcriptional control in group 3 medulloblastoma. A. Venn diagrams of ERCC-spike in normalized downregulated (left, blue) and upregulated (right, red) genes in HDMBO3 and MB002 cells after treatment with CCS1477, A485 or JQ1 for 6h, compared to DMSO controls. n=3 independent biological replicates. Intersection genes represent high-confidence down and upregulated targets. **B.** Gene set enrichment analysis comparing ERCC spike-in normalized transcriptomes of A485 or CCS1477 treated samples in MB002 cells, using the Hallmarks dataset identified the top differentially regulated gene set to be "Hallmarks_MYC_Targets_V2" (NES=1.82, FDR q-value of 9.45×10^{-4}). **C.** Normalized RNAseq gene expression of *c-MYC* in HDMBO3 and MB002 cells treated for 6h with DMSO, A485, CCS1477 or JQ1. *-$p<0.05$ by two-way ANOVA. **D.** METASCAPE analysis of top 10 enriched transcription factor binding sites in high-confidence genes downregulated after 6h treatment with CCS1477, A485 or JQ1 in HDMBO3 and MB002 cells. Highlighted in red are *c-MYC* relevant binding sites. **E.** Pie charts of relative ratios of dependent and non-dependent genes in high-confidence downregulated genes in HDMBO3 and MB002 cells after treatment with CCS1477, A485 or JQ1 for 6h. Dependency was determined using the 22Q4 release of Depmap, profiling CRISPR-cas9 dropout screening data in seven MB cell lines. **F,G.** String-database analysis of high-confidence A485 **(F)** and JQ1 **(G)**-downregulated gene dependencies in medulloblastoma cell lines. Line width indicates strength of known protein-protein interactions. Red indicates gene ontologies associated with transcription and cell cycle, blue indicates nucleic acid metabolism, grey indicates other.

Cell Line	Lineage Association	CCS1477 (AUC)	A485 (AUC)	Ratio CCS1477/A485
NUDHL1	Lymphoma	0.521789439	0.921279173	0.566374943
C32	Melanoma	0.587857056	0.933927252	0.629446303
MOLM13	AML	0.381416019	0.577618938	0.660324644
TTC1240	Rhabdoid	0.449734769	0.6274356	0.716782358
OC316	Ovarian Cancer	0.664026003	0.91874219	0.722755534
EB1	Lymphoma	0.748880762	1	0.748880762
BICR18	Head and Neck Cancer	0.753422811	1	0.753422811
EFE184	Endometrial/Uterine Cancer	0.769810173	1	0.769810173
143B	Osteosarcoma	0.775640748	1	0.775640748
NCIH650	Lung Cancer	0.641915815	0.815934372	0.786724812
HEC50B	Endometrial/Uterine Cancer	0.790118221	1	0.790118221
SHP77	Lung Cancer	0.790976616	1	0.790976616
SKNO1	AML	0.643240547	0.812588377	0.791594571
HCC2108	Lung Cancer	0.79489717	1	0.79489717
SNUC2A	Colon/Colorectal Cancer	0.80278539	1	0.80278539
BEN	Lung Cancer	0.712347014	0.88689631	0.803190865
SH10TC	Gastric Cancer	0.763670041	0.948334098	0.805275317
KMS12BM	Myeloma	0.808326466	0.997316937	0.810501092
MKN7	Gastric Cancer	0.776696839	0.950062435	0.817521891
TC71	Ewing Sarcoma	0.759906694	0.926229234	0.820430478
JHUEM7	Endometrial/Uterine Cancer	0.823901637	1	0.823901637
ONS76	Medulloblastoma	0.660204509	0.796920076	0.828445071
EBC1	Lung Cancer	0.731721482	0.873515627	0.837674175
G402	Rhabdoid	0.839375091	0.997530927	0.841452699
LK2	Lung Cancer	0.713234986	0.846971789	0.84210005
HDMYZ	Lymphoma	0.842292109	1	0.842292109
SNU478	Bile Duct Cancer	0.639161808	0.755648716	0.845845158
BT16	Rhabdoid	0.848060173	1	0.848060173
LOVO	Colon/Colorectal Cancer	0.631194167	0.739156736	0.853938193
NCIH647	Lung Cancer	0.854379067	1	0.854379067
OC314	Ovarian Cancer	0.763429083	0.890956245	0.856864843
SNU201	Glioblastoma	0.711992125	0.825886496	0.862094402
HT29	Colon/Colorectal Cancer	0.657977023	0.762949531	0.862412251
KMS28BM	Myeloma	0.70578692	0.816980526	0.863896872
OCIAML2	AML	0.532374272	0.615871156	0.864424754
RDES	Ewing Sarcoma	0.562890196	0.650674226	0.865087587
SKNBE2	Neuroblastoma	0.620766252	0.711035068	0.873045901
RHJT	Rhabdomyosarcoma	0.876374096	1	0.876374096
COLO320	Colon/Colorectal Cancer	0.883119052	1	0.883119052
G401	Rhabdoid	0.887481664	1	0.887481664
NCIH441	Lung Cancer	0.782600756	0.881768958	0.887534936
RH28	Rhabdomyosarcoma	0.887692554	1	0.887692554
T47D	Breast Cancer	0.879105958	0.970400674	0.905920597
MELHO	Melanoma	0.722441823	0.794416414	0.909399416

MOLT16	T-acute leukemia	0.846503981	0.927330006	0.912840062
SNU1	Gastric Cancer	0.913085147	1	0.913085147
NCIH1341	Lung Cancer	0.913559526	1	0.913559526
HEPG2	Liver Cancer	0.788232135	0.855424688	0.921451235
K029AX	Melanoma	0.732733375	0.793261483	0.923697154
NCIH2170	Lung Cancer	0.875472189	0.943364795	0.928031439
SKM1	AML	0.62131562	0.6690963	0.928589232
FADU	Head and Neck Cancer	0.891842378	0.957304619	0.931618171
SKNDZ	Neuroblastoma	0.814314122	0.872713134	0.933083382
G361	Melanoma	0.634860453	0.680287907	0.933223193
NCIH1930	Lung Cancer	0.933787889	1	0.933787889
M059K	Glioblastoma	0.811330416	0.867742296	0.934990054
U2OS	Osteosarcoma	0.862305211	0.921673194	0.935586732
CHAGOK1	Lung Cancer	0.877070085	0.937154338	0.935886491
SW1116	Colon/Colorectal Cancer	0.898488188	0.954680386	0.941140303
HT115	Colon/Colorectal Cancer	0.941275506	1	0.941275506
AM38	Glioblastoma	0.942172228	1	0.942172228
SNU1214	Head and Neck Cancer	0.730671645	0.771264048	0.947368994
RH36	Rhabdomyosarcoma	0.955764314	1	0.955764314
HT1197	Bladder Cancer	0.948480633	0.982923906	0.964958352
TTC442	Rhabdomyosarcoma	0.842506293	0.871607225	0.966612332
HEC1B	Endometrial/Uterine Cancer	0.839343272	0.866224936	0.968966878
ONCODG1	Ovarian Cancer	0.944239388	0.973395099	0.970047403
UW228	Medulloblastoma	0.970405572	1	0.970405572
LOXIMVI	Melanoma	0.868336198	0.892597317	0.972819637
SKNMC	Ewing Sarcoma	0.854631228	0.877884642	0.973511993
A673	Ewing Sarcoma	0.973965837	1	0.973965837
MHHNB11	Neuroblastoma	0.700345251	0.718180479	0.975166093
UBLC1	Bladder Cancer	0.694968672	0.712445933	0.975468649
GSS	Gastric Cancer	0.645987126	0.657856267	0.981957851
CHL1	Melanoma	0.611097233	0.620490029	0.984862293
CBAGPN	Ewing Sarcoma	0.822426756	0.834279001	0.985793428
PF382	T-acute leukemia	0.757096688	0.767407158	0.986564538
MOLM16	AML	0.986751169	1	0.986751169
SNU719	Gastric Cancer	0.91395337	0.925547784	0.987472917
SAOS2	Osteosarcoma	0.676605892	0.684290702	0.988769671
CAL78	Chondrosarcoma	0.869132235	0.877387887	0.990590647
OCILY132	T-cell lymphoma	0.927325779	0.935671929	0.991080046
SKUT1	Leiomyosarcoma	0.832578393	0.839748881	0.991461151
MKN1	Gastric Cancer	0.960555928	0.96712948	0.993203027
SKMEL30	Melanoma	0.798361809	0.803462546	0.993651556
HEYA8	Ovarian Cancer	0.972606845	0.978432125	0.994046312
OVCAR8	Ovarian Cancer	0.88578927	0.888925241	0.996472177
NCIH82	Lung Cancer	0.996966229	1	0.996966229
NCIH2172	Lung Cancer	0.846323826	0.848422209	0.997526723
COGE352	Ewing Sarcoma	0.998035156	1	0.998035156

KYSE410	Esophageal Cancer	0.954922187	0.956387524	0.998467842
NCIH2196	Lung Cancer	0.872493578	0.873368325	0.998998421
CHLA06ATRT	Rhabdoid	1	1	1
COGAR359	Rhabdoid	1	1	1
G292CLONEA141B1	Osteosarcoma	1	1	1
HUH7	Liver Cancer	1	1	1
MDAMB468	Breast Cancer	1	1	1
NB4	AML	1	1	1
SW48	Colon/Colorectal Cancer	1	1	1
HEL	AML	0.591436222	0.59136045	1.000128131
NIHOVCAR3	Ovarian Cancer	0.996721745	0.996413702	1.000309152
HCC1438	Lung Cancer	0.902739073	0.901449248	1.001430834
BT474	Breast Cancer	0.951195066	0.94981643	1.001451476
KMS34	Myeloma	0.872509243	0.868217942	1.004942654
NCIH1648	Lung Cancer	0.780227471	0.775987351	1.005464162
NCIH596	Lung Cancer	0.901115445	0.895417683	1.006363245
KMS21BM	Myeloma	0.942663781	0.933812857	1.009478263
BDCM	AML	0.911316258	0.90255222	1.009710283
HOS	Osteosarcoma	0.962799567	0.951660965	1.01170438
OE19	Esophageal Cancer	0.950803013	0.939178961	1.012376824
OVKATE	Ovarian Cancer	0.879121504	0.866284514	1.014818445
TE9	Esophageal Cancer	0.99812009	0.981873032	1.016547005
SNU449	Liver Cancer	0.950857171	0.934328652	1.017690262
OV56	Ovarian Cancer	1	0.98223833	1.018082851
STM9101	Rhabdoid	0.906096661	0.889959934	1.018131971
COLO792	Melanoma	0.807574489	0.792912011	1.018491936
VMRCRCW	Kidney Cancer	0.915943633	0.898741493	1.019140253
HEC6	Endometrial/Uterine Cancer	0.992915369	0.974073188	1.019343701
LCLC103H	Lung Cancer	0.944983293	0.926436588	1.0200194
NCIH196	Lung Cancer	0.841969569	0.825024272	1.020539151
HEL9217	AML	0.624893977	0.611928164	1.021188456
NCIH1568	Lung Cancer	0.941152723	0.920956048	1.021930118
GP2D	Colon/Colorectal Cancer	0.774788052	0.757486882	1.022840224
PK59	Pancreatic Cancer	1	0.977174423	1.023358755
GB1	Glioblastoma	0.776745812	0.758162448	1.024511059
OSRC2	Kidney Cancer	1	0.975787223	1.024813582
NCIH2004RT	Rhabdoid	1	0.974125197	1.026562092
T3M10	Lung Cancer	0.927886951	0.903643933	1.026828065
EN	Endometrial/Uterine Cancer	1	0.973550984	1.027167571
CHLA15	Neuroblastoma	0.739659326	0.71882132	1.028989133
PATU8902	Pancreatic Cancer	0.974381389	0.946514674	1.029441398
LUDLU1	Lung Cancer	0.967690001	0.939923074	1.029541702
NCIH460	Lung Cancer	0.993544952	0.964757922	1.029838605
OVISE	Ovarian Cancer	0.980293399	0.950973878	1.030831047
KPNYN	Neuroblastoma	0.962621792	0.933758353	1.030911037
NUGC3	Gastric Cancer	0.905034147	0.877537125	1.031334312

GOS3	Glioblastoma	0.984036807	0.953442181	1.032088601
OCIAML3	AML	0.807546882	0.781872995	1.032836391
PANC0504	Pancreatic Cancer	0.873166552	0.844818211	1.033555552
RI1	B-cell Lymphoma	0.703411759	0.678133486	1.037276249
GI1	Glioblastoma	0.785475884	0.75699972	1.037617138
SIMA	Neuroblastoma	0.800250203	0.768868719	1.04081514
CHLA57	Ewing Sarcoma	0.998548194	0.958353682	1.041941208
MON	Rhabdoid	0.659821384	0.632736601	1.042805779
MIAPACA2	Pancreatic Cancer	0.939619644	0.900896328	1.042983099
CAOV3	Ovarian Cancer	0.847842509	0.81240245	1.043623772
NCIH520	Lung Cancer	0.995117303	0.95214106	1.045136424
SKMM2	Myeloma	0.955441927	0.913943636	1.045405745
KMS11	Myeloma	0.830454581	0.794360012	1.045438552
EM2	CML	0.943189053	0.902020927	1.045639879
MDAMB436	Breast Cancer	1	0.954428811	1.04774708
TE5	Esophageal Cancer	0.876715466	0.835496543	1.049334643
RL952	Endometrial/Uterine Cancer	0.993612634	0.94604074	1.05028525
HUT78	Lymphoma	0.992470151	0.944139834	1.051189788
KARPAS620	Myeloma	0.948563533	0.900208728	1.053715103
MDAMB361	Breast Cancer	0.740073281	0.701563605	1.054891211
MFE280	Endometrial/Uterine Cancer	0.820534166	0.777456128	1.055408963
SNU1041	Head and Neck Cancer	0.882781849	0.836016841	1.055937878
LOUNH91	Lung Cancer	1	0.946457391	1.0565716
CHP212	Neuroblastoma	0.853675502	0.807159894	1.05762874
SW620	Colon/Colorectal Cancer	1	0.945397927	1.057755651
CAL120	Breast Cancer	0.948189056	0.895066618	1.059350262
DL	Rhabdoid	0.741525777	0.699617893	1.059901104
SKHEP1	Liver Cancer	0.972610259	0.917639549	1.059904469
MC116	B-cell Lymphoma	0.937708288	0.882074227	1.063071858
SNU398	Liver Cancer	0.888794027	0.835834789	1.063360892
HCT116	Colon/Colorectal Cancer	0.979159078	0.920531262	1.063689109
HCC33	Lung Cancer	0.96688846	0.907989498	1.064867448
A204	Rhabdoid	0.693523638	0.651259833	1.064895458
TCCPAN2	Pancreatic Cancer	0.941358041	0.88395548	1.064938293
HGC27	Gastric Cancer	0.983141326	0.922561949	1.065664292
CW2	Colon/Colorectal Cancer	0.993890874	0.932434623	1.065909448
SQ1	Lung Cancer	0.890216826	0.834681431	1.06653484
BICR16	Head and Neck Cancer	0.727573367	0.680361561	1.069392231
JHH1	Liver Cancer	0.766415752	0.716135281	1.070210857
TOLEDO	Lymphoma	0.969700597	0.905116871	1.071354018
RERFLCAI	Lung Cancer	0.936965478	0.874038295	1.071995911
MESSA	Uterine Sarcoma	0.764594578	0.713185387	1.07208391
SNU869	Bile Duct Cancer	0.999369392	0.93152793	1.072828156
A101D	Melanoma	0.994865254	0.925837224	1.074557415
GCT	Pleomorphic Sarcoma	0.961203538	0.893295907	1.07601919
COLO783	Melanoma	0.973562336	0.904537242	1.076309842

SNU601	Gastric Cancer	0.894081166	0.82977028	1.077504446
T24	Bladder Cancer	0.996078695	0.923183417	1.078960775
OCILY19	Lymphoma	0.724753857	0.671054436	1.080022452
MDAMB175VII	Breast Cancer	0.999616841	0.924674886	1.081046816
A253	Head and Neck Cancer	0.889518188	0.822765761	1.081131751
EWS502	Ewing Sarcoma	0.710156005	0.656251823	1.082139477
SNU1196	Bile Duct Cancer	0.88734302	0.819069212	1.083355359
AN3CA	Endometrial/Uterine Cancer	0.94214256	0.869216508	1.083898604
HPBALL	T-acute leukemia	0.95155378	0.876715274	1.085362384
AGS	Gastric Cancer	0.851977607	0.784865062	1.085508386
MCF7	Breast Cancer	0.791274672	0.728728106	1.08582977
ISHIKAWAHERAKLIO02ER	Endometrial/Uterine Cancer	0.997140461	0.917829016	1.086412004
NCO2	CML	0.912188563	0.835053378	1.092371562
RD	Rhabdomyosarcoma	0.895437223	0.818788248	1.093612697
SNU685	Endometrial/Uterine Cancer	0.960055054	0.876586654	1.095219793
KP3	Pancreatic Cancer	1	0.911757951	1.096782319
MG63	Osteosarcoma	1	0.91148141	1.09711508
EFO21	Ovarian Cancer	0.990141028	0.902386305	1.097247401
KD	Rhabdoid	0.705154235	0.64253735	1.097452521
5637	Bladder Cancer	0.970929597	0.884296076	1.097968908
CORL23	Lung Cancer	0.912489776	0.831010562	1.098048349
ESS1	Endometrial/Uterine Cancer	0.996612565	0.904398614	1.101961624
NCIH1944	Lung Cancer	0.962362637	0.872925922	1.102456248
KMBC2	Bladder Cancer	0.801217346	0.726079056	1.103484999
SNU668	Gastric Cancer	0.959097005	0.868850392	1.103868991
TE10	Esophageal Cancer	0.779218447	0.705515888	1.104466193
SW780	Bladder Cancer	0.869393745	0.786703547	1.105109732
U266B1	Myeloma	0.986648737	0.892515874	1.105469119
OVK18	Ovarian Cancer	0.596882867	0.539690444	1.105972644
SNU245	Bile Duct Cancer	0.836643003	0.756410711	1.106069747
SNU1079	Bile Duct Cancer	0.993954829	0.897554667	1.107403109
SNU1077	Endometrial/Uterine Cancer	0.994831969	0.897769507	1.108115125
CAL62	Thyroid Cancer	0.956421489	0.8629336	1.108337291
MDAMB453	Breast Cancer	0.575938204	0.518182253	1.111458759
SNU878	Liver Cancer	0.796547632	0.71632415	1.111993267
TE4	Esophageal Cancer	1	0.898617794	1.112820164
KNS42	Glioblastoma	0.925127797	0.830424484	1.114042053
MDST8	Colon/Colorectal Cancer	0.916163048	0.822298662	1.114148777
COLO678	Colon/Colorectal Cancer	0.884799963	0.793841638	1.114579936
JHOC5	Ovarian Cancer	1	0.896862021	1.114998714
DMS53	Lung Cancer	0.712019243	0.638204593	1.115659853
SKNSH	Neuroblastoma	0.833587418	0.744084301	1.12028626
NCIH841	Lung Cancer	0.972068757	0.866297541	1.122095713
DU145	Prostate Cancer	0.950139131	0.84633758	1.122647928
PANC0327	Pancreatic Cancer	1	0.888957044	1.124913748
VMCUB1	Bladder Cancer	0.743671884	0.661056275	1.124975153

GRANTA519	Lymphoma	0.957131768	0.849346039	1.126904376
SNU1076	Head and Neck Cancer	0.892899302	0.792240887	1.127055315
OVTOKO	Ovarian Cancer	0.932771379	0.826216162	1.128967723
TOV21G	Ovarian Cancer	0.992410911	0.878593818	1.12954461
HCC95	Lung Cancer	0.9968086	0.882290662	1.129796158
HCC515	Lung Cancer	0.780259874	0.690142157	1.130578484
SCC9	Head and Neck Cancer	0.789578168	0.698234347	1.130821151
HCC78	Lung Cancer	0.897233618	0.793142655	1.13123864
HUPT4	Pancreatic Cancer	0.777069252	0.68617337	1.132467808
42MGBA	Glioma	0.958973195	0.846773359	1.132502794
HT	B-cell Lymphoma	0.70782105	0.624714245	1.133031713
DKMG	Glioblastoma	1	0.881674223	1.134205781
JHH4	Liver Cancer	0.964103957	0.849013149	1.135558334
KYSE150	Esophageal Cancer	0.987969468	0.868119917	1.138056447
SCC25	Head and Neck Cancer	0.984938062	0.86372683	1.140335148
BICR22	Head and Neck Cancer	0.861812085	0.755606652	1.140556509
YD10B	Head and Neck Cancer	0.867873386	0.760776026	1.140773837
DAOY	Medulloblastoma	0.887056078	0.777341804	1.141140324
SNU61	Colon/Colorectal Cancer	0.934639454	0.818666381	1.141660969
CCK81	Colon/Colorectal Cancer	0.822684525	0.719507367	1.143399725
MDAMB231	Breast Cancer	0.881139964	0.770319736	1.143862636
KU1919	Bladder Cancer	0.736147691	0.643117558	1.144654943
SNU213	Pancreatic Cancer	0.985311996	0.860107459	1.145568482
YAPC	Pancreatic Cancer	1	0.872634084	1.145955697
LS411N	Colon/Colorectal Cancer	0.973243184	0.849203524	1.146065879
CL11	Colon/Colorectal Cancer	0.760956368	0.6639168	1.146162242
J82	Bladder Cancer	1	0.870682822	1.148523865
IGROV1	Ovarian Cancer	0.960056012	0.835862136	1.14858177
HCC1806	Breast Cancer	0.983134602	0.854819568	1.15010774
IM95	Gastric Cancer	0.819427852	0.711712131	1.151347317
KNS62	Lung Cancer	0.951555182	0.826429787	1.151404749
HH	Lymphoma	0.894798421	0.776394214	1.152505268
CORL105	Lung Cancer	0.878194874	0.761621994	1.153058709
PANC0813	Pancreatic Cancer	1	0.865487446	1.155418261
U937	AML	0.795704605	0.687536497	1.157327078
YD38	Head and Neck Cancer	0.689818001	0.595917341	1.157573297
ECC10	Gastric Cancer	0.97762149	0.844415771	1.157748971
TCCSUP	Bladder Cancer	0.981974181	0.848069258	1.157893852
TYKNU	Ovarian Cancer	0.850192324	0.73413351	1.15808952
NCIH1048	Lung Cancer	1	0.863104647	1.15860806
TT	Thyroid Cancer	0.769488864	0.663731073	1.159338315
KMS18	Myeloma	0.888088672	0.765729834	1.159793745
HS294T	Melanoma	0.965922698	0.832601083	1.160126641
NCIH358	Lung Cancer	1	0.861106398	1.161296679
KCL22	CML	0.729315955	0.62752291	1.162214069
RCM1	Colon/Colorectal Cancer	0.958570057	0.824514734	1.162586934

SKNEP1	Ewing Sarcoma	0.802497937	0.690155468	1.162778495
FTC133	Thyroid Cancer	0.960130651	0.825419054	1.163203886
NB1	Neuroblastoma	0.573686118	0.492973683	1.163725647
COV644	Ovarian Cancer	0.876764403	0.7527539	1.164742424
NCIH1838	Lung Cancer	0.963916288	0.82746045	1.164909195
HT144	Melanoma	1	0.857367207	1.166361381
SCLC21H	Lung Cancer	0.873295003	0.748233475	1.167142385
SNU423	Liver Cancer	0.916153352	0.784638929	1.167611391
IPC298	Melanoma	0.938776412	0.803343712	1.168586244
NCIH28	Lung Cancer	0.992759653	0.848840336	1.16954816
LNCAPCLONEFGC	Prostate Cancer	0.718324823	0.611883595	1.173956662
WM793	Melanoma	0.997762207	0.849688708	1.174267937
TE1	Esophageal Cancer	1	0.851580666	1.174286876
NCIH226	Lung Cancer	0.987378738	0.84024281	1.175111202
LI7	Liver Cancer	0.84686646	0.720279926	1.175746302
NCIH23	Lung Cancer	0.977142306	0.83069164	1.176299675
UACC257	Melanoma	0.963771449	0.819279607	1.176364504
BHT101	Thyroid Cancer	0.721623805	0.613305358	1.176614218
PC3	Prostate Cancer	1	0.84935199	1.177368172
ECGI10	Esophageal Cancer	0.99529718	0.843478562	1.179991082
NCIH1581	Lung Cancer	0.908061293	0.769363078	1.180276671
NCIH1299	Lung Cancer	0.994745882	0.842357354	1.180907221
HUH1	Liver Cancer	0.846763557	0.71577447	1.183003297
S117	Thyroid Sarcoma	0.869712767	0.733034772	1.186454996
NCIH292	Lung Cancer	0.931916549	0.784065137	1.188570318
WM983B	Melanoma	0.841219128	0.707056037	1.189748881
KS1	Glioma	0.792114042	0.66533004	1.190558061
NCIH747	Colon/Colorectal Cancer	0.998110152	0.838026686	1.191024306
C8166	Lymphoma	0.824589323	0.691550673	1.192377298
HCC38	Breast Cancer	0.886768344	0.743490751	1.192709314
CI1	B-cell Lymphoma	0.92780309	0.777858165	1.192766408
RERFLCKJ	Lung Cancer	0.882682452	0.739266183	1.193998146
HSC3	Head and Neck Cancer	0.976902033	0.817609359	1.194827361
JHH5	Liver Cancer	0.681766856	0.570056958	1.195962695
SR786	Lymphoma	0.790459936	0.660932929	1.195976023
A375	Melanoma	0.895961022	0.748952967	1.196284762
IALM	Lung Cancer	0.785561717	0.65594036	1.197611499
KE37	T-acute leukemia	0.804876975	0.671573684	1.198493917
SNU81	Colon/Colorectal Cancer	1	0.834359902	1.19852356
SNU1105	Glioblastoma	0.987216431	0.823047523	1.199464677
NCIH524	Lung Cancer	1	0.831724836	1.202320715
NCIH2444	Lung Cancer	1	0.83160869	1.202488637
RH4	Rhabdomyosarcoma	0.816274506	0.67804644	1.20386224
RT4	Bladder Cancer	0.972126298	0.80684505	1.20484881
SNU216	Gastric Cancer	0.976853533	0.809612398	1.206569384
639V	Bladder Cancer	1	0.827432724	1.208557471

NCIH1792	Lung Cancer	0.835207755	0.691011101	1.208674873
HUCCT1	Bile Duct Cancer	0.990501014	0.815765886	1.214197641
MOLT3	T-acute leukemia	0.992368652	0.816152282	1.215911141
HUH28	Bile Duct Cancer	0.999054128	0.821002802	1.216870546
COV318	Ovarian Cancer	0.898976211	0.736618864	1.220408891
BICR31	Head and Neck Cancer	0.86535738	0.707741622	1.222702401
NCIH1975	Lung Cancer	0.992668896	0.811454542	1.223320401
SNU466	Glioblastoma	0.999253464	0.815947471	1.224654158
A427	Lung Cancer	0.853363409	0.696802069	1.224685527
AU565	Breast Cancer	0.973404026	0.794420719	1.225300402
ISTMES1	Lung Cancer	0.928174111	0.757380939	1.225504979
YD15	Head and Neck Cancer	0.767859093	0.625925234	1.226758488
SW900	Lung Cancer	0.953816028	0.773953674	1.232394212
SKES1	Ewing Sarcoma	0.701540035	0.568644048	1.23370681
SH4	Melanoma	0.925153789	0.749070204	1.235069536
TE15	Esophageal Cancer	0.858618906	0.689839359	1.244665001
SW1088	Glioma	0.9903592	0.795151096	1.245498127
YH13	Glioblastoma	0.96728754	0.775128259	1.247906431
NCIH1573	Lung Cancer	1	0.800470589	1.249265137
UACC62	Melanoma	0.896428729	0.716955526	1.250326828
ES2	Ovarian Cancer	0.935478169	0.747711194	1.251122327
DND41	T-acute leukemia	0.939460261	0.750214962	1.252254764
EFM19	Breast Cancer	0.75122199	0.599245184	1.253613729
RPMI8402	T-acute leukemia	0.815254563	0.648826556	1.256506158
HUPT3	Pancreatic Cancer	0.868688595	0.689458534	1.259957709
PSN1	Pancreatic Cancer	0.778253279	0.617158766	1.261026047
CHLA10	Ewing Sarcoma	0.981419169	0.778121209	1.261267726
RL	B-cell Lymphoma	0.890451439	0.705634669	1.261915659
CMLT1	CML	0.834251736	0.660732895	1.262615713
KNS60	Glioblastoma	0.894191369	0.707190512	1.264427836
HEP3B217	Liver Cancer	0.915096409	0.723393624	1.265004804
NCIH1793	Lung Cancer	0.960924227	0.757199768	1.269049818
SJSA1	Osteosarcoma	0.880980153	0.693793976	1.26980081
HS766T	Pancreatic Cancer	0.972154471	0.764804199	1.271115498
HCC1428	Breast Cancer	0.812330779	0.638099699	1.273046798
NCIH211	Lung Cancer	0.868703563	0.682365255	1.273077075
SW1463	Colon/Colorectal Cancer	0.886358441	0.695800209	1.273869179
HUG1N	Gastric Cancer	0.949998593	0.744798444	1.275510979
SCABER	Bladder Cancer	0.926348037	0.72540706	1.277004442
NCIH2228	Lung Cancer	1	0.781085729	1.280269198
SNU1066	Head and Neck Cancer	1	0.780930162	1.280524238
KYSE510	Esophageal Cancer	0.928028629	0.724691333	1.280584694
LAMA84	CML	0.887439135	0.692755218	1.281028438
HDQP1	Breast Cancer	0.939015168	0.729435789	1.287317105
DMS273	Lung Cancer	1	0.776216395	1.28830054
UMUC1	Bladder Cancer	0.831914206	0.645438817	1.288912573

A1207	Glioblastoma	1	0.771189758	1.296697718
TTC642	Rhabdoid	0.784352102	0.603951475	1.298700533
SKOV3	Ovarian Cancer	0.997531671	0.767952979	1.298948891
SF539	Glioma	0.991239854	0.762628513	1.299767629
SW579	Thyroid Cancer	0.995740053	0.765863856	1.300152821
NCIH322	Lung Cancer	0.992890664	0.761584219	1.303717486
8305C	Thyroid Cancer	0.984225942	0.754444275	1.304570761
CADOES1	Ewing Sarcoma	0.898553855	0.686527735	1.30883839
SKNAS	Neuroblastoma	0.978929729	0.74653576	1.3112965
TE617T	Rhabdomyosarcoma	0.994739119	0.757464905	1.313247799
NCIH1373	Lung Cancer	0.974130288	0.741292362	1.314097295
MALME3M	Melanoma	0.798793932	0.604804373	1.320747612
BICR56	Head and Neck Cancer	0.894236084	0.676880933	1.321112829
CAL27	Head and Neck Cancer	0.998239574	0.754473616	1.32309408
LS180	Colon/Colorectal Cancer	0.98727032	0.745260183	1.324732413
HCC1419	Breast Cancer	0.816197263	0.616053235	1.32488106
FTC238	Thyroid Cancer	0.996143036	0.748412032	1.331008847
SW837	Colon/Colorectal Cancer	0.899565672	0.675047305	1.332596494
SNU182	Liver Cancer	0.773473674	0.580165886	1.333193993
TTC549	Rhabdoid	0.913069106	0.683038664	1.336775141
22RV1	Prostate Cancer	0.782370215	0.583443154	1.340953629
HARA	Lung Cancer	0.961177187	0.710832683	1.352184853
SNU407	Colon/Colorectal Cancer	0.992270634	0.733328911	1.35310448
EW8	Ewing Sarcoma	1	0.737067063	1.356728649
TC205	Ewing Sarcoma	0.755818188	0.553975276	1.364353645
769P	Kidney Cancer	0.866032213	0.633177675	1.367755445
OAW42	Ovarian Cancer	0.996846311	0.7266105	1.37191289
LMSU	Gastric Cancer	0.99181283	0.722283858	1.373162116
HCC4006	Lung Cancer	0.970300959	0.702413269	1.381381877
MCAS	Ovarian Cancer	0.995176436	0.72023003	1.381748045
JEKO1	Lymphoma	0.97959986	0.707338448	1.384909675
PANC0203	Pancreatic Cancer	0.960897231	0.69282479	1.386926745
VMRCRCZ	Kidney Cancer	0.827747391	0.59677558	1.387032947
SNU886	Liver Cancer	1	0.720686277	1.387566314
KE39	Gastric Cancer	1	0.720346805	1.388220219
SNUC4	Colon/Colorectal Cancer	0.894023657	0.641632662	1.393357462
LN18	Glioblastoma	0.990513331	0.70970914	1.395660948
SKMES1	Lung Cancer	0.95808039	0.685830388	1.396964041
PECAPJ15	Head and Neck Cancer	0.929482284	0.665257218	1.3971773
SW1710	Bladder Cancer	1	0.715702356	1.397228878
CW9019	Rhabdomyosarcoma	0.902098544	0.645231753	1.398100047
HSC2	Head and Neck Cancer	0.969500334	0.689590272	1.405907789
JVM3	B-cell Leukemia	0.948074068	0.674271156	1.406072409
647V	Bladder Cancer	0.938719976	0.667154607	1.40705013
KATOIII	Gastric Cancer	0.978271386	0.695076253	1.407430309
KYSE520	Esophageal Cancer	0.995291346	0.705513543	1.410733154

CJM	Melanoma	0.884174281	0.626633445	1.410991207
TALL1	T-acute leukemia	0.986445552	0.698509416	1.412215111
LXF289	Lung Cancer	0.97278642	0.688713474	1.412468982
LC1SQSF	Lung Cancer	0.993715295	0.702678895	1.414181216
CL34	Colon/Colorectal Cancer	0.942663652	0.665390137	1.416708182
NCIH2052	Lung Cancer	0.994092684	0.699703575	1.420734036
OE21	Esophageal Cancer	0.99433952	0.699786557	1.420918293
SNU46	Head and Neck Cancer	0.922402386	0.648474433	1.422419048
SW948	Colon/Colorectal Cancer	0.80652464	0.566690173	1.423219738
TE6	Esophageal Cancer	0.974421011	0.68452826	1.423492742
PK1	Pancreatic Cancer	0.993686863	0.697357412	1.424931959
TM87	Rhabdoid	0.958111036	0.672273669	1.425180072
DEL	Lymphoma	1	0.700674804	1.427195603
BFTC905	Bladder Cancer	0.962862508	0.671537793	1.433817305
HCC1937	Breast Cancer	1	0.697140923	1.43443021
SEM	B-cell Leukemia	0.851014857	0.5909545	1.440068324
PANC0403	Pancreatic Cancer	0.993799258	0.689444398	1.441449463
KALS1	Glioblastoma	0.98997953	0.686580552	1.441898589
HCC44	Lung Cancer	0.945557813	0.655755484	1.44193657
CAKI1	Kidney Cancer	0.990611185	0.684923197	1.446309878
TT2609C02	Thyroid Cancer	0.879825776	0.607765427	1.447640383
JHH7	Liver Cancer	0.981848122	0.677878237	1.448413696
OCIM1	AML	0.897667628	0.618481393	1.451406038
TF1	AML	0.666796441	0.457278811	1.458183551
IGR37	Melanoma	1	0.684323381	1.461297433
NCIH1437	Lung Cancer	0.993181477	0.672374435	1.477125579
DETROIT562	Head and Neck Cancer	0.917719145	0.61893597	1.482736809
DV90	Lung Cancer	0.915379961	0.612756942	1.493871221
NCIH661	Lung Cancer	1	0.667711146	1.497653598
697	B-cell Leukemia	0.842119986	0.561704607	1.499222147
COV434	Ovarian Cancer	0.968727	0.639217067	1.515489887
HCC56	Colon/Colorectal Cancer	0.96053402	0.621186431	1.546289441
RVH421	Melanoma	0.974980243	0.62611461	1.557191332
TE11	Esophageal Cancer	0.925758552	0.591485689	1.565141084
WM88	Melanoma	0.917723346	0.581625632	1.577859186
KYSE30	Esophageal Cancer	0.989005007	0.625591836	1.580910988
MKN45	Gastric Cancer	0.855961694	0.523537354	1.634958208
KELLY	Neuroblastoma	0.860926871	0.52424603	1.642219152
NCIH2347	Lung Cancer	0.998916302	0.603855081	1.654231839
KYM1	Rhabdoid	0.94752937	0.562204869	1.685380941
SUDHL8	Lymphoma	0.898287236	0.529530291	1.696384988
ACHN	Kidney Cancer	0.930304139	0.541730829	1.717281147
TC106	Ewing Sarcoma	1	0.580686791	1.722098756
CAL54	Kidney Cancer	0.839060777	0.481283886	1.743380158
DU4475	Breast Cancer	0.748350535	0.423308892	1.767859236
LS1034	Colon/Colorectal Cancer	0.877108435	0.481928627	1.819996544

NCIH2122	Lung Cancer	0.933421611	0.480214201	1.943760949
MHHES1	Ewing Sarcoma	0.918898874	0.455279307	2.018318997

Supplementary Table 1. Area under the curve measurements for CCS1477 and A485 treated cell lines.

Cell Line	Lineage Association	CCS1477 (AUC)	A485 (AUC)	Ratio CCS1477/A485
NUDHL1	Lymphoma	0.56905448	1.160956916	0.490159861
C32	Melanoma	0.641106674	1.176895489	0.54474393
MOLM13	AML	0.415965672	0.727890872	0.571467081
TTC1240	Rhabdoid	0.490472911	0.790667715	0.620327479
OC316	Ovarian Cancer	0.724175201	1.157759918	0.625496866
EB1	Lymphoma	0.816716325	1.260157562	0.648106514
BICR18	Head and Neck Cancer	0.821669804	1.260157562	0.652037356
EFE184	Endometrial/Uterine Cancer	0.839541576	1.260157562	0.666219528
143B	Osteosarcoma	0.845900299	1.260157562	0.671265503
NCIH650	Lung Cancer	0.700062215	1.028205869	0.680858023
HEC50B	Endometrial/Uterine Cancer	0.861689179	1.260157562	0.683794793
SHP77	Lung Cancer	0.86262533	1.260157562	0.684537677
SKNO1	AML	0.701506945	1.023989388	0.685072476
HCC2108	Lung Cancer	0.866901018	1.260157562	0.687930656
SNUC2A	Colon/Colorectal Cancer	0.875503773	1.260157562	0.694757386
BEN	Lung Cancer	0.776873255	1.117629091	0.695108297
SH10TC	Gastric Cancer	0.832845255	1.195050384	0.696912253
KMS12BM	Myeloma	0.881546775	1.25677648	0.701434813
MKN7	Gastric Cancer	0.847052053	1.197228361	0.707510848
TC71	Ewing Sarcoma	0.828741014	1.167194774	0.710028037
JHUEM7	Endometrial/Uterine Cancer	0.898532785	1.260157562	0.713032094
ONS76	Medulloblastoma	0.720007546	1.004244859	0.716964134
EBC1	Lung Cancer	0.798002713	1.100767323	0.72495131
G402	Rhabdoid	0.915407864	1.257046141	0.728221371
LK2	Lung Cancer	0.777841661	1.067317904	0.72878161
HDMYZ	Lymphoma	0.918589113	1.260157562	0.728947825
SNU478	Bile Duct Cancer	0.697058743	0.952236444	0.732022753
BT16	Rhabdoid	0.924879664	1.260157562	0.733939701
LOVO	Colon/Colorectal Cancer	0.688369372	0.93145395	0.739026736
NCIH647	Lung Cancer	0.93177094	1.260157562	0.739408284
OC314	Ovarian Cancer	0.83258247	1.12274525	0.741559557
SNU201	Glioblastoma	0.776486219	1.040747113	0.746085393
HT29	Colon/Colorectal Cancer	0.717578288	0.961436621	0.74636047
KMS28BM	Myeloma	0.769718931	1.029524188	0.747645311
OCIAML2	AML	0.580598115	0.776094694	0.748102158
RDES	Ewing Sarcoma	0.61387825	0.819952046	0.748675796
SKNBE2	Neuroblastoma	0.676996869	0.896016218	0.755563187
RHJT	Rhabdomyosarcoma	0.955758336	1.260157562	0.758443519
COLO320	Colon/Colorectal Cancer	0.963114267	1.260157562	0.76428083
G401	Rhabdoid	0.967872056	1.260157562	0.768056381
NCIH441	Lung Cancer	0.853490763	1.111167821	0.768102484
RH28	Rhabdomyosarcoma	0.968102049	1.260157562	0.768238892

T47D	Breast Cancer	0.958737656	1.222857748	0.784014051
MELHO	Melanoma	0.787882478	1.001089851	0.787024738
MOLT16	T-acute leukemia	0.923182508	1.16858192	0.790002388
SNU1	Gastric Cancer	0.995794768	1.260157562	0.790214493
NCIH1341	Lung Cancer	0.996312118	1.260157562	0.790625037
HEPG2	Liver Cancer	0.859632247	1.077969889	0.797454786
K029AX	Melanoma	0.799106265	0.999634457	0.79939848
NCIH2170	Lung Cancer	0.954774731	1.188788281	0.803149515
SKM1	AML	0.677596001	0.843166763	0.803632248
FADU	Head and Neck Cancer	0.972627775	1.206354655	0.806253593
SKNDZ	Neuroblastoma	0.888076808	1.099756055	0.807521636
G361	Melanoma	0.69236776	0.85726995	0.807642633
NCIH1930	Lung Cancer	1.018372819	1.260157562	0.80813134
M059K	Glioblastoma	0.884822831	1.093492016	0.809171734
U2OS	Osteosarcoma	0.940415055	1.161453445	0.809688118
CHAGOK1	Lung Cancer	0.956517369	1.180962126	0.80994754
SW1116	Colon/Colorectal Cancer	0.97987558	1.203047708	0.814494365
HT115	Colon/Colorectal Cancer	1.026538683	1.260157562	0.814611374
AM38	Glioblastoma	1.027516633	1.260157562	0.815387428
SNU1214	Head and Neck Cancer	0.796857778	0.971914222	0.819884883
RH36	Rhabdomyosarcoma	1.042339925	1.260157562	0.827150474
HT1197	Bladder Cancer	1.034396469	1.238638992	0.835107304
TTC442	Rhabdomyosarcoma	0.918822698	1.098362436	0.836538713
HEC1B	Endometrial/Uterine Cancer	0.915373163	1.091579904	0.838576416
ONCODG1	Ovarian Cancer	1.029771042	1.226631195	0.839511538
UW228	Medulloblastoma	1.058307427	1.260157562	0.83982151
LOXIMVI	Melanoma	0.946992343	1.124813258	0.841910723
SKNMC	Ewing Sarcoma	0.932045942	1.10627297	0.842509911
A673	Ewing Sarcoma	1.062190189	1.260157562	0.842902682
MHHNB11	Neuroblastoma	0.763784341	0.905020561	0.843941424
UBLC1	Bladder Cancer	0.757920738	0.89779413	0.844203267
GSS	Gastric Cancer	0.704502316	0.829002549	0.84981924
CHL1	Melanoma	0.666452006	0.781915203	0.852332841
CBAGPN	Ewing Sarcoma	0.896924305	1.051322991	0.853138677
PF382	T-acute leukemia	0.825676471	0.967053933	0.853806021
MOLM16	AML	1.07613365	1.260157562	0.853967538
SNU719	Gastric Cancer	0.996741637	1.166336039	0.854592163
SAOS2	Osteosarcoma	0.737894609	0.862314102	0.855714417
CAL78	Chondrosarcoma	0.947860487	1.105646981	0.85729035
OCILY132	T-cell lymphoma	1.011325354	1.179094057	0.857713893
SKUT1	Leiomyosarcoma	0.907995504	1.058215903	0.858043714
MKN1	Gastric Cancer	1.047565576	1.218735528	0.859551192
SKMEL30	Melanoma	0.870679493	1.012489403	0.859939364
HEYA8	Ovarian Cancer	1.060708097	1.232978641	0.860280999
OVCAR8	Ovarian Cancer	0.96602636	1.120185864	0.862380424

NCIH82	Lung Cancer	1.087274016	1.260157562	0.862807993
NCIH2172	Lung Cancer	0.922986034	1.069145662	0.863293063
COGE352	Ewing Sarcoma	1.08843977	1.260157562	0.863733078
KYSE410	Esophageal Cancer	1.041421516	1.20519897	0.864107539
NCIH2196	Lung Cancer	0.95152631	1.100581699	0.86456672
CHLA06ATRT	Rhabdoid	1.090582595	1.260157562	0.86543352
COGAR359	Rhabdoid	1.090582595	1.260157562	0.86543352
G292CLONEA141B1	Osteosarcoma	1.090582595	1.260157562	0.86543352
HUH7	Liver Cancer	1.090582595	1.260157562	0.86543352
MDAMB468	Breast Cancer	1.090582595	1.260157562	0.86543352
NB4	AML	1.090582595	1.260157562	0.86543352
SW48	Colon/Colorectal Cancer	1.090582595	1.260157562	0.86543352
HEL	AML	0.645010049	0.745207343	0.865544409
NIHOVCAR3	Ovarian Cancer	1.087007386	1.255638261	0.86570107
HCC1438	Lung Cancer	0.98451152	1.135968087	0.866671812
BT474	Breast Cancer	1.037356783	1.196918357	0.866689676
KMS34	Myeloma	0.951543394	1.094091405	0.869711059
NCIH1648	Lung Cancer	0.8509025	0.977866328	0.870162389
NCIH596	Lung Cancer	0.98274082	1.128367365	0.870940485
KMS21BM	Myeloma	1.028052712	1.176751333	0.873636327
BDCM	AML	0.993865649	1.137358005	0.873837125
HOS	Osteosarcoma	1.050012449	1.199242762	0.875562883
OE19	Esophageal Cancer	1.036929217	1.183513469	0.876144838
OVKATE	Ovarian Cancer	0.95875461	1.091654981	0.878257899
TE9	Esophageal Cancer	1.088532397	1.237314727	0.879753852
SNU449	Liver Cancer	1.036988281	1.177401317	0.880743266
OV56	Ovarian Cancer	1.090582595	1.23777506	0.881083026
STM9101	Rhabdoid	0.988173248	1.12148974	0.881125536
COLO792	Melanoma	0.880726682	0.999194067	0.881437061
VMRCRCW	Kidney Cancer	0.998912183	1.132555888	0.881998137
HEC6	Endometrial/Uterine Cancer	1.082856219	1.227485694	0.882174207
LCLC103H	Lung Cancer	1.030582331	1.167456072	0.88275898
NCIH196	Lung Cancer	0.918237358	1.039660575	0.883208789
HEL9217	AML	0.681498495	0.771125903	0.88377072
NCIH1568	Lung Cancer	1.026404778	1.160549728	0.88441258
GP2D	Colon/Colorectal Cancer	0.844970364	0.954552823	0.885200215
PK59	Pancreatic Cancer	1.090582595	1.231393738	0.885648969
GB1	Glioblastoma	0.847105463	0.955404142	0.886646212
OSRC2	Kidney Cancer	1.090582595	1.229645648	0.886908026
NCIH2004RT	Rhabdoid	1.090582595	1.227551233	0.888421245
T3M10	Lung Cancer	1.011937359	1.138733736	0.888651427
EN	Endometrial/Uterine Cancer	1.090582595	1.226827634	0.888945247
CHLA15	Neuroblastoma	0.806659587	0.905828122	0.890521687
PATU8902	Pancreatic Cancer	1.062643383	1.192757623	0.890913093

LUDLU1	Lung Cancer	1.055345872	1.184451169	0.890999899
NCIH460	Lung Cancer	1.083542832	1.21574699	0.891256849
OVISE	Ovarian Cancer	1.069090918	1.198376924	0.892115742
KPNYN	Neuroblastoma	1.049818572	1.176682649	0.892184968
NUGC3	Gastric Cancer	0.987014488	1.105835043	0.892551284
GOS3	Glioblastoma	1.073173414	1.201487374	0.893204071
OCIAML3	AML	0.880696574	0.985283167	0.893851233
PANC0504	Pancreatic Cancer	0.952260244	1.064604057	0.89447362
RI1	B-cell Lymphoma	0.767128621	0.854555041	0.897693636
GI1	Glioblastoma	0.856626327	0.953938922	0.897988653
SIMA	Neuroblastoma	0.872738943	0.96889573	0.90075631
CHLA57	Ewing Sarcoma	1.08899928	1.20767664	0.901730848
MON	Rhabdoid	0.719589717	0.797347813	0.902479076
MIAPACA2	Pancreatic Cancer	1.024732829	1.13527132	0.902632535
CAOV3	Ovarian Cancer	0.924642283	1.023755091	0.903186994
NCIH520	Lung Cancer	1.08525761	1.199847757	0.904496095
SKMM2	Myeloma	1.041988336	1.151712983	0.904729174
KMS11	Myeloma	0.905679312	1.001018776	0.904757566
EM2	CML	1.028625565	1.136688492	0.904931801
MDAMB436	Breast Cancer	1.090582595	1.202730683	0.906755444
TE5	Esophageal Cancer	0.956130628	1.052857286	0.908129374
RL952	Endometrial/Uterine Cancer	1.083616645	1.192160392	0.908952061
HUT78	Lymphoma	1.082370673	1.189764951	0.909734878
KARPAS620	Myeloma	1.034486879	1.134404836	0.911920371
MDAMB361	Breast Cancer	0.807111038	0.884080682	0.912938214
MFE280	Endometrial/Uterine Cancer	0.894860279	0.979717218	0.913386294
SNU1041	Head and Neck Cancer	0.962746519	1.053512944	0.913844035
LOUNH91	Lung Cancer	1.090582595	1.192685438	0.914392479
CHP212	Neuroblastoma	0.931003644	1.017148644	0.915307363
SW620	Colon/Colorectal Cancer	1.090582595	1.191350347	0.915417196
CAL120	Breast Cancer	1.034078481	1.127924967	0.916797226
DL	Rhabdoid	0.808695105	0.881628778	0.917273943
SKHEP1	Liver Cancer	1.06071182	1.156370417	0.917276855
MC116	B-cell Lymphoma	1.022648338	1.111552508	0.92001802
SNU398	Liver Cancer	0.969303295	1.05328353	0.92026816
HCT116	Colon/Colorectal Cancer	1.067853847	1.16001443	0.92055221
HCC33	Lung Cancer	1.054471725	1.144209832	0.921571984
A204	Rhabdoid	0.756344809	0.820690003	0.921596225
TCCPAN2	Pancreatic Cancer	1.026628694	1.113923183	0.921633296
HGC27	Gastric Cancer	1.072196818	1.162573416	0.922261599
CW2	Colon/Colorectal Cancer	1.083920088	1.175014542	0.922473765
SQ1	Lung Cancer	0.970854976	1.051830117	0.923015001
BICR16	Head and Neck Cancer	0.79347885	0.857362765	0.925487883
JHH1	Liver Cancer	0.83583968	0.902443289	0.926196349

TOLEDO	Lymphoma	1.057538593	1.14058987	0.927185679
RERFLCAI	Lung Cancer	1.021838242	1.101425967	0.927741194
MESSA	Uterine Sarcoma	0.833853538	0.898725958	0.927817352
SNU869	Bile Duct Cancer	1.089894864	1.173871965	0.928461448
A101D	Melanoma	1.08498273	1.166700778	0.929958006
GCT	Pleomorphic Sarcoma	1.048271848	1.125693592	0.931223075
COLO783	Melanoma	1.061750138	1.139859445	0.931474615
SNU601	Gastric Cancer	0.975069357	1.045641293	0.932508465
T24	Bladder Cancer	1.086306088	1.163356564	0.933768822
OCILY19	Lymphoma	0.790403942	0.845634322	0.934687632
MDAMB175VII	Breast Cancer	1.090164728	1.16523605	0.935574151
A253	Head and Neck Cancer	0.970093053	1.036814495	0.935647657
EWS502	Ewing Sarcoma	0.774483778	0.826980698	0.936519777
SNU1196	Bile Duct Cancer	0.967720853	1.032156261	0.937572042
AN3CA	Endometrial/Uterine Cancer	1.027484277	1.095349756	0.938042184
HPBALL	T-acute leukemia	1.037747991	1.104799382	0.939308989
AGS	Gastric Cancer	0.929151949	0.989053643	0.939435343
MCF7	Breast Cancer	0.862950385	0.918312234	0.93971348
ISHIKAWAHERAKLIO02ER	Endometrial/Uterine Cancer	1.087464031	1.156609175	0.940217365
NCO2	CML	0.994816969	1.052298829	0.945374966
RD	Rhabdomyosarcoma	0.97654825	1.031802202	0.946449086
SNU685	Endometrial/Uterine Cancer	1.047019331	1.104637301	0.94783992
KP3	Pancreatic Cancer	1.090582595	1.148958676	0.949192183
MG63	Osteosarcoma	1.090582595	1.148610191	0.949480165
EFO21	Ovarian Cancer	1.079830571	1.137148926	0.949594681
KD	Rhabdoid	0.769028935	0.809698301	0.949772198
5637	Bladder Cancer	1.058878919	1.114352387	0.950219097
CORL23	Lung Cancer	0.995145467	1.047204244	0.950287848
ESS1	Endometrial/Uterine Cancer	1.086888317	1.139684752	0.953674527
NCIH1944	Lung Cancer	1.049535942	1.100024202	0.954102592
KMBC2	Bladder Cancer	0.873793692	0.914974013	0.954992907
SNU668	Gastric Cancer	1.0459745	1.094888391	0.955325226
TE10	Esophageal Cancer	0.849802075	0.889061181	0.955842065
SW780	Bladder Cancer	0.948145686	0.991370423	0.956399005
U266B1	Myeloma	1.076021939	1.124710627	0.956710031
OVK18	Ovarian Cancer	0.650950066	0.680094994	0.957145798
SNU245	Bile Duct Cancer	0.912428297	0.953196677	0.957229834
SNU1079	Bile Duct Cancer	1.083989836	1.131060301	0.958383771
SNU1077	Endometrial/Uterine Cancer	1.08494643	1.131331033	0.958999973
CAL62	Thyroid Cancer	1.043056629	1.087432301	0.959192244
MDAMB453	Breast Cancer	0.628108181	0.652991284	0.961893666
SNU878	Liver Cancer	0.868700983	0.902681294	0.962356247

TE4	Esophageal Cancer	1.090582595	1.132400008	0.963071871
KNS42	Glioblastoma	1.008928273	1.046465693	0.964129335
MDST8	Colon/Colorectal Cancer	0.999151474	1.036225877	0.964221698
COLO678	Colon/Colorectal Cancer	0.964947439	1.000365543	0.964594838
JHOC5	Ovarian Cancer	1.090582595	1.130187458	0.964957261
DMS53	Lung Cancer	0.776515793	0.804238344	0.965529434
SKNSH	Neuroblastoma	0.90909593	0.937663458	0.969533282
NCIH841	Lung Cancer	1.060121267	1.091671397	0.971099243
DU145	Prostate Cancer	1.036205198	1.066518702	0.971577148
PANC0327	Pancreatic Cancer	1.090582595	1.120225941	0.973538065
VMCUB1	Bladder Cancer	0.811035613	0.833035064	0.973591207
GRANTA519	Lymphoma	1.043831247	1.070309833	0.975260821
SNU1076	Head and Neck Cancer	0.973780438	0.998348345	0.975391448
OVTOKO	Ovarian Cancer	1.01726423	1.041162544	0.977046511
TOV21G	Ovarian Cancer	1.082306066	1.107166643	0.977545768
HCC95	Lung Cancer	1.087102109	1.11182525	0.977763466
HCC515	Lung Cancer	0.850937838	0.869687858	0.978440517
SCC9	Head and Neck Cancer	0.861100207	0.879885293	0.978650529
HCC78	Lung Cancer	0.978507367	0.999484714	0.979011838
HUPT4	Pancreatic Cancer	0.847458201	0.864686561	0.980075602
42MGBA	Glioma	1.045839475	1.067067852	0.98010588
HT	B-cell Lymphoma	0.771937318	0.787238379	0.980563623
DKMG	Glioblastoma	1.090582595	1.111048439	0.981579701
JHH4	Liver Cancer	1.051434995	1.06989034	0.982750246
KYSE150	Esophageal Cancer	1.077462306	1.093967878	0.984912197
SCC25	Head and Neck Cancer	1.074156307	1.088431896	0.986884261
BICR22	Head and Neck Cancer	0.93987726	0.952183436	0.987075835
YD10B	Head and Neck Cancer	0.94648761	0.958697662	0.987263918
DAOY	Medulloblastoma	0.967407919	0.979573153	0.987581087
SNU61	Colon/Colorectal Cancer	1.01930152	1.03164863	0.988031671
CCK81	Colon/Colorectal Cancer	0.897205424	0.906692649	0.989536449
MDAMB231	Breast Cancer	0.960955908	0.97072424	0.989937068
KU1919	Bladder Cancer	0.802829859	0.810429453	0.990622756
SNU213	Pancreatic Cancer	1.074564113	1.083870918	0.991413364
YAPC	Pancreatic Cancer	1.090582595	1.09965644	0.991748472
LS411N	Colon/Colorectal Cancer	1.061402077	1.070130243	0.991843828
CL11	Colon/Colorectal Cancer	0.82988577	0.836639776	0.991927223
J82	Bladder Cancer	1.090582595	1.097197542	0.993971052
IGROV1	Ovarian Cancer	1.047020376	1.053317991	0.994021165
HCC1806	Breast Cancer	1.072189484	1.077207343	0.99534179
IM95	Gastric Cancer	0.893653753	0.896869423	0.996414561
KNS62	Lung Cancer	1.037749519	1.041431745	0.996464265
HH	Lymphoma	0.975851584	0.97837904	0.997416691
CORL105	Lung Cancer	0.957744044	0.959763716	0.997895657
PANC0813	Pancreatic Cancer	1.090582595	1.09065055	0.999937693
U937	AML	0.867781593	0.866404316	1.001589647

YD38	Head and Neck Cancer	0.752303505	0.750949743	1.001802733
ECC10	Gastric Cancer	1.066176981	1.064096919	1.001954767
TCCSUP	Bladder Cancer	1.07092395	1.068700889	1.002080153
TYKNU	Ovarian Cancer	0.92720495	0.925123893	1.00224949
NCIH1048	Lung Cancer	1.090582595	1.087647847	1.002698251
TT	Thyroid Cancer	0.839191161	0.83640573	1.003330239
KMS18	Myeloma	0.968534048	0.96494024	1.003724383
HS294T	Melanoma	1.053418482	1.049208551	1.004012482
NCIH358	Lung Cancer	1.090582595	1.085129739	1.005025073
KCL22	CML	0.795379287	0.790777741	1.005819013
RCM1	Colon/Colorectal Cancer	1.04539982	1.039018477	1.006141703
SKNEP1	Ewing Sarcoma	0.875190282	0.869704632	1.006307486
FTC133	Thyroid Cancer	1.047101776	1.040158063	1.006675633
NB1	Neuroblastoma	0.625652095	0.621224515	1.007127183
COV644	Ovarian Cancer	0.956183997	0.94858852	1.008007136
NCIH1838	Lung Cancer	1.051230326	1.042730544	1.008151466
HT144	Melanoma	1.090582595	1.080417769	1.009408236
SCLC21H	Lung Cancer	0.95240033	0.942892072	1.010084143
SNU423	Liver Cancer	0.999140899	0.98876868	1.010490037
IPC298	Melanoma	1.023813215	1.012339654	1.011333707
NCIH28	Lung Cancer	1.082686398	1.069672568	1.012166181
LNCAPCLONEFGC	Prostate Cancer	0.783392549	0.771069739	1.015981447
WM793	Melanoma	1.088142096	1.070741651	1.016250835
TE1	Esophageal Cancer	1.090582595	1.073125816	1.016267224
NCIH226	Lung Cancer	1.076818066	1.05883833	1.016980624
LI7	Liver Cancer	0.923577821	0.907666196	1.017530261
NCIH23	Lung Cancer	1.065654391	1.046802351	1.018009168
UACC257	Melanoma	1.051072367	1.032421392	1.018065273
BHT101	Thyroid Cancer	0.786990361	0.772861385	1.018281384
PC3	Prostate Cancer	1.090582595	1.070317333	1.018933882
ECGI10	Esophageal Cancer	1.085453781	1.062915888	1.021203835
NCIH1581	Lung Cancer	0.990315841	0.969518701	1.021450994
NCIH1299	Lung Cancer	1.084852545	1.061502989	1.021996693
HUH1	Liver Cancer	0.923465597	0.90198861	1.023810707
S117	Thyroid Sarcoma	0.948493606	0.92373931	1.026797924
NCIH292	Lung Cancer	1.016331968	0.988045611	1.028628594
WM983B	Melanoma	0.91741894	0.891002011	1.029648562
KS1	Glioma	0.863865787	0.83842068	1.030348854
NCIH747	Colon/Colorectal Cancer	1.088521559	1.056045665	1.030752358
C8166	Lymphoma	0.899282764	0.871462811	1.031923282
HCC38	Breast Cancer	0.967094122	0.936915493	1.03221062
CI1	B-cell Lymphoma	1.011845901	0.980223849	1.032260031
RERFLCKJ	Lung Cancer	0.962638118	0.93159187	1.033326019
HSC3	Head and Neck Cancer	1.065392354	1.030316617	1.034043649
JHH5	Liver Cancer	0.743523066	0.718361586	1.035026205
SR786	Lymphoma	0.862061848	0.832879628	1.03503774

A375	Melanoma	0.977119496	0.943798745	1.035304932
IALM	Lung Cancer	0.856719935	0.826588204	1.036453135
KE37	T-acute leukemia	0.87778482	0.846288656	1.03721681
SNU81	Colon/Colorectal Cancer	1.090582595	1.051424939	1.037242464
SNU1105	Glioblastoma	1.076641057	1.03716956	1.038056937
NCIH524	Lung Cancer	1.090582595	1.048104342	1.040528649
NCIH2444	Lung Cancer	1.090582595	1.047957979	1.040673974
RH4	Rhabdomyosarcoma	0.890214769	0.854445349	1.041862736
RT4	Bladder Cancer	1.06018402	1.016751891	1.042716547
SNU216	Gastric Cancer	1.06533946	1.020239186	1.044205589
639V	Bladder Cancer	1.090582595	1.042695603	1.045926147
NCIH1792	Lung Cancer	0.91086304	0.870782865	1.04602775
HUCCT1	Bile Duct Cancer	1.080223166	1.02799355	1.050807338
MOLT3	T-acute leukemia	1.082259979	1.028480469	1.052290258
HUH28	Bile Duct Cancer	1.089551042	1.034592889	1.05312056
COV318	Ovarian Cancer	0.980407808	0.928255831	1.056182763
BICR31	Head and Neck Cancer	0.943743696	0.891865956	1.058167643
NCIH1975	Lung Cancer	1.08258742	1.022560577	1.058702481
SNU466	Glioblastoma	1.089768435	1.028222376	1.059856759
A427	Lung Cancer	0.930663281	0.878080396	1.059883906
AU565	Breast Cancer	1.061577489	1.001095276	1.06041604
ISTMES1	Lung Cancer	1.012250531	0.954419317	1.060593088
YD15	Head and Neck Cancer	0.837413762	0.788764416	1.061677916
SW900	Lung Cancer	1.040215158	0.975303574	1.066555261
SKES1	Ewing Sarcoma	0.765087352	0.716581098	1.067691227
SH4	Melanoma	1.008956619	0.943946481	1.068870576
TE15	Esophageal Cancer	0.936394834	0.869306284	1.077174813
SW1088	Glioma	1.080068506	1.002015666	1.077895828
YH13	Glioblastoma	1.054906955	0.976783737	1.079980056
NCIH1573	Lung Cancer	1.090582595	1.008719066	1.081155925
UACC62	Melanoma	0.977629569	0.903476927	1.082074748
ES2	Ovarian Cancer	1.020216209	0.942233915	1.0827632
DND41	T-acute leukemia	1.024559009	0.945389057	1.083743249
EFM19	Breast Cancer	0.819269626	0.75514335	1.084919342
RPMI8402	T-acute leukemia	0.889102437	0.817623691	1.087422547
HUPT3	Pancreatic Cancer	0.947376662	0.868826385	1.090409636
PSN1	Pancreatic Cancer	0.84874948	0.777717286	1.091334211
CHLA10	Ewing Sarcoma	1.070318663	0.980555326	1.091543368
RL	B-cell Lymphoma	0.97111084	0.889210864	1.092104111
CMLT1	CML	0.909820423	0.832627554	1.092709961
KNS60	Glioblastoma	0.975189543	0.891171471	1.094278233
HEP3B217	Liver Cancer	0.997988216	0.911589945	1.09477756
NCIH1793	Lung Cancer	1.047967237	0.954191013	1.098278251
SJSA1	Osteosarcoma	0.960781621	0.874289725	1.098928185
HS766T	Pancreatic Cancer	1.060214745	0.963773795	1.100065959
HCC1428	Breast Cancer	0.885913809	0.804106161	1.101737372

NCIH211	Lung Cancer	0.947392985	0.859887736	1.101763575
SW1463	Colon/Colorectal Cancer	0.966647089	0.876817895	1.102449087
HUG1N	Gastric Cancer	1.03605193	0.938563392	1.103869957
SCABER	Bladder Cancer	1.010259046	0.914127192	1.105162449
NCIH2228	Lung Cancer	1.090582595	0.984291088	1.107987879
SNU1066	Head and Neck Cancer	1.090582595	0.984095048	1.108208599
KYSE510	Esophageal Cancer	1.01209187	0.913225263	1.108260919
LAMA84	CML	0.967825674	0.872980726	1.10864495
HDQP1	Breast Cancer	1.024073598	0.919204025	1.114087374
DMS273	Lung Cancer	1.090582595	0.978154959	1.114938471
UMUC1	Bladder Cancer	0.907271153	0.813354605	1.115468145
A1207	Glioblastoma	1.090582595	0.971820606	1.122205671
TTC642	Rhabdoid	0.85540075	0.761074018	1.123938974
SKOV3	Ovarian Cancer	1.087890677	0.967741754	1.124153911
SF539	Glioma	1.081028932	0.961032087	1.124862474
SW579	Thyroid Cancer	1.08593677	0.965109129	1.125195832
NCIH322	Lung Cancer	1.082829276	0.959716113	1.128280813
8305C	Thyroid Cancer	1.073379681	0.950718658	1.129019266
CADOES1	Ewing Sarcoma	0.979947195	0.865133116	1.132712615
SKNAS	Neuroblastoma	1.067603724	0.940752683	1.134839946
TE617T	Rhabdomyosarcoma	1.084845169	0.954525128	1.136528665
NCIH1373	Lung Cancer	1.062369537	0.934145176	1.137263848
MALME3M	Melanoma	0.871150759	0.762148805	1.143019255
BICR56	Head and Neck Cancer	0.975238309	0.852976627	1.143335326
CAL27	Head and Neck Cancer	1.088662705	0.950755632	1.145049967
LS180	Colon/Colorectal Cancer	1.076699827	0.939145255	1.146467835
HCC1419	Breast Cancer	0.890130528	0.776324142	1.146596479
FTC238	Thyroid Cancer	1.086376257	0.943117082	1.151899672
SW837	Colon/Colorectal Cancer	0.981050664	0.850665966	1.153273674
SNU182	Liver Cancer	0.843536926	0.731100429	1.15379077
TTC549	Rhabdoid	0.995777275	0.860736337	1.156890016
22RV1	Prostate Cancer	0.853239339	0.735230303	1.160506219
HARA	Lung Cancer	1.04824311	0.89576118	1.170226097
SNU407	Colon/Colorectal Cancer	1.082153083	0.924109972	1.171021973
EW8	Ewing Sarcoma	1.090582595	0.928820633	1.17415845
TC205	Ewing Sarcoma	0.82428216	0.698096134	1.180757378
769P	Kidney Cancer	0.944479657	0.797903635	1.183701409
OAW42	Ovarian Cancer	1.087143236	0.915643716	1.187299402
LMSU	Gastric Cancer	1.08165381	0.910191465	1.188380523
HCC4006	Lung Cancer	1.058193338	0.885151392	1.19549418
MCAS	Ovarian Cancer	1.0853221	0.907603319	1.195811075
JEKO1	Lymphoma	1.068334557	0.891357894	1.198547255
PANC0203	Pancreatic Cancer	1.047937796	0.873068398	1.200292895
VMRCRCZ	Kidney Cancer	0.902726897	0.752031259	1.200384806
SNU886	Liver Cancer	1.090582595	0.908178261	1.200846399
KE39	Gastric Cancer	1.090582595	0.907750474	1.201412311

SNUC4	Colon/Colorectal Cancer	0.975006639	0.80855825	1.205858253
LN18	Glioblastoma	1.080236598	0.894345339	1.207851767
SKMES1	Lung Cancer	1.044865798	0.86425435	1.208979507
PECAPJ15	Head and Neck Cancer	1.013677201	0.838328914	1.209164069
SW1710	Bladder Cancer	1.090582595	0.901897736	1.209208706
CW9019	Rhabdomyosarcoma	0.983812971	0.813093673	1.209962645
HSC2	Head and Neck Cancer	1.05732019	0.868992396	1.216719726
JVM3	B-cell Leukemia	1.033953077	0.849687895	1.216862194
647V	Bladder Cancer	1.023751667	0.840719922	1.217708347
KATOIII	Gastric Cancer	1.066885746	0.875905596	1.218037367
KYSE520	Esophageal Cancer	1.085447418	0.889058226	1.22089576
CJM	Melanoma	0.964265082	0.789656875	1.221119087
TALL1	T-acute leukemia	1.075800349	0.880231922	1.222178294
LXF289	Lung Cancer	1.060903938	0.867887492	1.222398003
LC1SQSF	Lung Cancer	1.083728604	0.885486123	1.223879828
CL34	Colon/Colorectal Cancer	1.028052572	0.838496413	1.226066749
NCIH2052	Lung Cancer	1.084140179	0.881736751	1.229550858
OE21	Esophageal Cancer	1.084409373	0.881841321	1.22971032
SNU46	Head and Neck Cancer	1.005955987	0.81717996	1.231009124
SW948	Colon/Colorectal Cancer	0.879581734	0.714118907	1.231702068
TE6	Esophageal Cancer	1.062686594	0.862613463	1.231938335
PK1	Pancreatic Cancer	1.083697597	0.878780215	1.233183881
TM87	Rhabdoid	1.044899219	0.847170747	1.233398607
DEL	Lymphoma	1.090582595	0.882960653	1.235142915
BFTC905	Bladder Cancer	1.050081093	0.846243428	1.240873558
HCC1937	Breast Cancer	1.090582595	0.878507405	1.241403986
SEM	B-cell Leukemia	0.928101991	0.744695782	1.246283399
PANC0403	Pancreatic Cancer	1.083820173	0.868808572	1.247478682
KALS1	Glioblastoma	1.079654444	0.865199674	1.247867372
HCC44	Lung Cancer	1.031208893	0.826355232	1.247900242
CAKI1	Kidney Cancer	1.080343316	0.863111146	1.251685048
TT2609C02	Thyroid Cancer	0.959522677	0.765880199	1.252836513
JHH7	Liver Cancer	1.070786472	0.854233386	1.253505763
OCIM1	AML	0.97898069	0.779384004	1.256095437
TF1	AML	0.727196593	0.576243352	1.261960923
IGR37	Melanoma	1.090582595	0.862355283	1.264655781
NCIH1437	Lung Cancer	1.083146432	0.847297729	1.278353989
DETROIT562	Head and Neck Cancer	1.000848526	0.779956842	1.283210136
DV90	Lung Cancer	0.998297453	0.772170294	1.29284623
NCIH661	Lung Cancer	1.090582595	0.841421249	1.296119625
697	B-cell Leukemia	0.9184014	0.707836307	1.2974771
COV434	Ovarian Cancer	1.056476805	0.80551422	1.311555747
HCC56	Colon/Colorectal Cancer	1.047541683	0.782792779	1.338210714
RVH421	Melanoma	1.063296483	0.78900306	1.347645576
TE11	Esophageal Cancer	1.009616164	0.745365164	1.354525558
WM88	Melanoma	1.000853108	0.732939938	1.36553223

KYSE30	Esophageal Cancer	1.078591647	0.788344283	1.368173361
MKN45	Gastric Cancer	0.933496925	0.659739556	1.414947637
KELLY	Neuroblastoma	0.938911861	0.660632599	1.421231501
NCIH2347	Lung Cancer	1.089400732	0.760952547	1.431627683
KYM1	Rhabdoid	1.033359039	0.708466716	1.45858516
SUDHL8	Lymphoma	0.979656424	0.6672916	1.468108431
ACHN	Kidney Cancer	1.014573502	0.6826662	1.486192668
TC106	Ewing Sarcoma	1.090582595	0.73175685	1.490361988
CAL54	Kidney Cancer	0.915065079	0.606493528	1.508779627
DU4475	Breast Cancer	0.816138068	0.533435902	1.529964642
LS1034	Colon/Colorectal Cancer	0.956559193	0.607306004	1.575086015
NCIH2122	Lung Cancer	1.017973363	0.605145557	1.68219588
MHHES1	Ewing Sarcoma	1.002135118	0.573723661	1.746720914

Supplementary Table 2. Median normalized area under the curve measurements for CCS1477 and A485 treated cell lines

Compound	DiscoveRx Gene Symbol	Entrez Gene Symbol	Percent Control
CCS1477	ATAD2A	ATAD2	100
CCS1477	ATAD2B	ATAD2B	79
CCS1477	BAZ2A	BAZ2A	79
CCS1477	BAZ2B	BAZ2B	82
CCS1477	BRD1	BRD1	88
CCS1477	BRD2(1)	BRD2	3.5
CCS1477	BRD2(2)	BRD2	57
CCS1477	BRD3(1)	BRD3	1.7
CCS1477	BRD3(2)	BRD3	66
CCS1477	BRD4(1)	BRD4	3.3
CCS1477	BRD4(2)	BRD4	75
CCS1477	BRD7	BRD7	64
CCS1477	BRD9	BRD9	39
CCS1477	BRDT(1)	BRDT	14
CCS1477	BRDT(2)	BRDT	76
CCS1477	BRPF1	BRPF1	31
CCS1477	BRPF3	BRPF3	75
CCS1477	CECR2	CECR2	78
CCS1477	CREBBP	CREBBP	0.7
CCS1477	EP300	EP300	1.6
CCS1477	FALZ	BPTF	63
CCS1477	GCN5L2	KAT2A	100
CCS1477	PBRM1(2)	PBRM1	78
CCS1477	PBRM1(5)	PBRM1	71
CCS1477	PCAF	KAT2B	88
CCS1477	SMARCA2	SMARCA2	50
CCS1477	SMARCA4	SMARCA4	61
CCS1477	TAF1(2)	TAF1	69
CCS1477	TAF1L(2)	TAF1L	80
CCS1477	TRIM24(PHD,Bromo.)	TRIM24	54
CCS1477	TRIM33(PHD,Bromo.)	TRIM33	90
CCS1477	WDR9(2)	BRWD1	14

Supplementary Table 3. Bromoscan profiling of CCS1477 (1uM) across 32 human bromodomains

Treatment (6h)	Gene	# MB with CHRONOS score <-0.5	Gene Ontology	Annotation (2)
CCS1477	**DDX10**	7	RNA binding	RNA helicase
CCS1477	**DDX21**	7	RNA binding	RNA helicase
CCS1477	**DKC1**	7	RNA binding	
CCS1477	**EEF2KMT**	7	Enzyme	Lysine methyltransferase
CCS1477	**GEMIN5**	7	RNA binding	
CCS1477	**IMP4**	7	RNA binding	RNA metabolism
CCS1477	**IPO13**	7	Receptor/Transporter	
CCS1477	**MAK16**	7	RNA binding	Ribosomal subunit
CCS1477	**MYC**	7	Transcription	Transcription factor
CCS1477	**NIP7**	7	RNA binding	Ribosomal subunit
CCS1477	**NOL6**	7	RNA binding	RNA metabolism
CCS1477	**NUFIP1**	7	RNA binding	RNA metabolism
CCS1477	**PDCD2**	7	Unknown	
CCS1477	**POLR1B**	7	Transcription	RNA polymerase
CCS1477	**RRP1**	7	RNA binding	
CCS1477	**RRP9**	7	RNA binding	RNA metabolism
CCS1477	**TBCC**	7	Chaperone	cytoskeletal
CCS1477	**UTP15**	7	RNA binding	Regulation of transcription
CCS1477	**CDK6**	6	Cell cycle	Enzyme
CCS1477	**MARS2**	5	RNA binding	Translation
CCS1477	**SOWAHC**	5	Unknown	
CCS1477	**GLRX5**	4	Enzyme	
CCS1477	**INHBE**	4	Growth Factor	Signaling protein
CCS1477	**CD3EAP**	4	Transcription	RNA polymerase
CCS1477	**TRMT61A**	4	RNA binding	RNA methyltransferase
CCS1477	**NEUROD1**	3	Transcription	Transcription Factor
CCS1477	**PPAT**	3	Enzyme	DNA transferase
CCS1477	**SLC16A1**	3	Receptor/Transporter	
CCS1477	**AK4**	2	Nucleotide metabolism	Enzyme
CCS1477	**DDN**	2	Enzyme	
CCS1477	**DHODH**	2	Enzyme	
CCS1477	**KATNB1**	2	Cytoskeleton	
CCS1477	**LDB1**	2	Transcription	Transcription cofactor
CCS1477	**NFKBIE**	2	Membrane Trafficking	
CCS1477	**CCND3**	1	Cell Cycle	
CCS1477	**COBLL1**	1	Cytoskeleton	
CCS1477	**ESRRA**	1	Transcription	Nuclear receptor
CCS1477	**JMY**	1	Transcription	Transcription cofactor
CCS1477	**LRP8**	1	Receptor/Transporter	Cell surface receptor
CCS1477	**PAQR5**	1	Receptor/Transporter	GPCR
CCS1477	**PUS7**	1	RNA binding	Enzyme

CCS1477	**SOX2**	1	Transcription	Transcription factor

Treatment (6h)	Gene	# MB with CHRONOS score <-0.5	Gene Ontology	Annotation (2)
JQ1	**HMGCS1**	7	Enzyme	
JQ1	**HRK**	7	Apoptosis process	
JQ1	**MYBBP1A**	7	Chromatin binding	
JQ1	**RBMX**	7	RNA binding	RNA metabolism
JQ1	**RBMXL1**	7	RNA binding	RNA metabolism
JQ1	**SFPQ**	7	RNA binding	RNA metabolism
JQ1	**TTC27**	7	Unknown	
JQ1	**NDC1**	6	Nuclear pore	
JQ1	**PRDM10**	6	Transcription	Transcription Factor
JQ1	**RAD51D**	6	Enzyme	DNA metabolism
JQ1	**MVK**	5	Enzyme	
JQ1	**ARHGAP27**	4	Signal Transduction	GTPase activating protien
JQ1	**FAM136A**	4	Unknown	
JQ1	**INHBE**	4	Growth Factor	Signaling protein
JQ1	**PTPA**	4	Enzyme	
JQ1	**VPS52**	4	Membrane Trafficking	
JQ1	**ANKRD65**	3	Unknown	
JQ1	**NEUROD1**	3	Transcription	Transcription Factor
JQ1	**ZMYND8**	3	Transcription	Transcription Factor
JQ1	**DDN**	2	Enzyme	
JQ1	**LIN28B**	2	RNA binding	RNA metabolism
JQ1	**MRPL3**	2	RNA binding	ribosome
JQ1	**NFE2L3**	2	Transcription	Transcription Factor
JQ1	**OLFML3**	2	Signal Transduction	
JQ1	**SPRYD4**	2	Enzyme	ubiquitin-protein ligase
JQ1	**ADAM11**	1	Enzyme	Metalloprotease
JQ1	**CFLAR**	1	Apoptosis process	
JQ1	**COBLL1**	1	Cytoskeleton	
JQ1	**CROCC**	1	Chromatin binding	
JQ1	**GPR61**	1	Receptor/Transporter	GPCR
JQ1	**ITGA4**	1	Receptor/Transporter	Cell adhesion
JQ1	**LIG3**	1	DNA metabolism	DNA ligase
JQ1	**MEX3A**	1	RNA binding	RNA metabolism
JQ1	**SOX2**	1	Transcription	Transcription factor
JQ1	**TFAP4**	1	Transcription	Transcription Factor

Treatment (6h)	Gene	# MB with CHRONOS score <-0.5	Gene Ontology	Annotation (2)
A485	**DDX47**	7	RNA binding	RNA helicase
A485	**HMGCS1**	7	Enzyme	

A485	**IPO13**	7	Receptor/Transporter	
A485	**MYC**	7	Transcription	Transcription factor
A485	**RRP9**	7	RNA binding	RNA metabolism
A485	**TBCC**	7	Chaperone	cytoskeletal
A485	**SKP2**	5	Enzyme	Ubiquitin-protein ligase
A485	**SOWAHC**	5	Unknown	
A485	**CD3EAP**	4	Transcription	RNA polymerase
A485	**IGSF3**	3	Receptor/Transporter	
A485	**PGM5**	2	Enzyme	
A485	**ABHD15**	1	Enzyme	
A485	**CCND3**	1	Cell Cycle	
A485	**KCNH6**	1	Receptor/Transporter	
A485	**LRP8**	1	Receptor/Transporter	Cell surface receptor
A485	**PAQR5**	1	Receptor/Transporter	GPCR
A485	**PUS7**	1	RNA binding	Enzyme
A485	**SLC10A7**	1	Receptor/Transporter	
A485	**TLE3**	1	Transcription	Transcription cofactor
A485	**UNC119**	1	Membrane Trafficking	

Supplementary Table 4. PANTHER annotations of high confidence downregulated gene targets.

Protein	CBP	EP300	BRD4-BD1
**Inhibitor**	**CCS1447**	**CCS1447**	**CCS1447**
**PDB ID**	8FV2	8FVF	8FVK
**Ligand ID**	JHL	JHL	JHL
**Wavelength (Å)**	1.5418	1.0000	0.9700
**Resolution range (Å)**	41.43 - 1.87<br>(1.90 - 1.87)	19.74 - 2.10<br>(2.20 - 2.10)	34.20 - 1.53<br>(1.57 - 1.53)
**Space group**	P 41	P 4 21 2	P 21 21 21
**Unit cell**<br>a, b, c<br>α, β, γ	79.45 79.45 97.13<br>90.0 90.0 90.0	103.15 103.15 59.22<br>90.0 90.0 90.0	42.00 54.81 117.83<br>90.0 90.0 90.0
**Unique reflections**	49406 (2111)	19185 (2449)	41819 (2997)
**Completeness (%)**	99.1 (84.0)	99.8 (100)	99.7 (97.4)
**Mean I/sigma(I)**	9.9 (0.8)	16.0 (3.5)	21.5 (3.2)
**Wilson B-factor (Å^2)**	17.8	28.2	20.9
**R-meas**	0.155 (0.966)	0.175 (0.88)	0.060 (0.503)
**CC (1/2)**	0.985 (0.535)	0.998 (0.891)	0.999 (0.900)
**R-work (%)**	19.0 (34.3)	18.1 (19.8)	16.1
**R-free (%)**	22.6 (32.3)	23.9 (25.1)	18.9
**# of R-free reflections**	1489	1056	1099
**RMSD (bonds)**	0.008	0.008	0.009
**RMSD (angles)**	1.11	1.157	1.107
**Ramachandran (%)**			
favored	99.34	100	100
allowed	0.66	0.0	0.0
outliers	0.0	0.0	0.0
**Average B-factor (Å^2)**	21.7	24.6	17.9
protein	20.9	24.1	16.4
ligands	21.2	22.7	16.6
water	27.1	28.3	26.5
Values in parenthesis are for the highest resolution bins.			

Supplementary Table 5. Crystallographic data collection and refinement statistics.

Protein	CBP				
Inhibitor	CCS1477int (1)	iCBP4 (4)	iCBP5 (5)	iCBP6 (6)	iCBP8 (7)
PDB ID	8FVS	8FXA	8GA2	8FXE	8FXO
Ligand ID	YBE	YJY	YVK	YID	YN5
Wavelength (Å)	1.0000	1.0000	1.0332	1.0000	1.0000
Resolution range (Å)	39.79 - 1.75 (1.80 - 1.75)	39.59 - 1.65 (1.69 - 1.65)	46.05 - 1.85 (1.90 - 1.85)	40.37 - 1.55 (1.59 - 1.55)	32.34 - 1.74 (1.79 - 1.74)
Space group	P 1 21 1	P 1 21 1	P 1 21 1	P 21 21 21	C 1 2 1
Unit cell<br>a, b, c<br>α, β, γ	35.07 79.19 46.13 90.0 93.85 90.0	33.71 81.83 48.00 90.0 109.51 90.0	45.86 107.01 55.83 90.0 113.98 90.0	35.77 49.54 80.73 90.0 90.0 90.0	90.54 34.63 40.44 90.0 93.3 90 0
Unique reflections	25279 (1817)	27710 (1574)	41493 (2829)	21029 (1290)	12730 (842)
Completeness (%)	99.6 (98.8)	93.6 (71.4)	98.7 (91.9)	97.8 (83.7)	97.4 (88.9)
Mean I/sigma(I)	16.2 (6.1)	26.4 (15.2)	16.0 (4.6)	25.6 (7.5)	26.8 (7.6)
Wilson B-factor (Å^2)	18.6	18.9	36.1	23.2	30.0
R-meas	0.244 (0.574)	0.048 (0.084)	0.082 (0.666)	0.053 (0.202)	0.034 (0.13)
CC (1/2)	0.994 (0.931)	0.998 (0.995)	0.998 (0.844)	0.999 (0.975)	0.999 (0.992)
R-work (%)	15.2 (17.9)	15.7 (16.8)	19.1 (25.0)	15.6 (16.3)	17.6 (24.5)
R-free (%)	18.1 (19.8)	18.4 (21.5)	22.5 (33.5)	18.6 (23.4)	20.5 (30.1)
# of R-free reflections	1263	1386	2075	1052	1018
RMSD (bonds)	0.007	0.007	0.008	0.018	0.007
RMSD (angles)	1.113	1.129	1.16	1.694	1.042
Ramachandran (%)					
favored	100	99.12	99.78	100	99.11
allowed	0.0	0.88	0.22	0.0	0.89
outliers	0.0	0.0	0	0.0	0
Average B-factor (Å^2)	14.7	13.6	33.1	20.1	29.7
protein	13.1	12.4	33.2	18.5	28.9
ligands	15.0	15.0	30.4	18.3	31.8
water	23.4	20.6	32.9	29.8	35.3
Values in parenthesis are for the highest resolution bins.					

Supplementary Table 6. Crystallographic data collection and refinement statistics.

Supplementary References:

1. Welti, J. *et al.* Targeting the p300/CBP Axis in Lethal Prostate Cancer. *Cancer Discov* **11**, 1118-1137 (2021).
