# Supplementary material for: Group 3 medulloblastoma transcriptional networks collapse under domain specific EP300/CBP inhibition
Source: Nat Commun. 2024 Apr 25;15:3483. doi: 10.1038/s41467-024-47102-0 (PMC11045757; doi:10.1038/s41467-024-47102-0)
Supplement: Supplementary file 3 — Reporting Summary [file 41467_2024_47102_MOESM3_ESM.pdf]

nature portfolio

Corresponding author(s): Durbin AD, Qi J, Schonnbrun E

Last updated by author(s): Feb 25, 2024

# Reporting Summary

Nature Portfolio wishes to improve the reproducibility of the work that we publish. This form provides structure for consistency and transparency in reporting. For further information on Nature Portfolio policies, see our Editorial Policies and the Editorial Policy Checklist.

## Statistics

For all statistical analyses, confirm that the following items are present in the figure legend, table legend, main text, or Methods section.

| n/a | Confirmed | |
|---|---|---|
| ☐ | ☒ | The exact sample size (*n*) for each experimental group/condition, given as a discrete number and unit of measurement |
| ☐ | ☒ | A statement on whether measurements were taken from distinct samples or whether the same sample was measured repeatedly |
| ☐ | ☒ | The statistical test(s) used AND whether they are one- or two-sided<br>*Only common tests should be described solely by name; describe more complex techniques in the Methods section.* |
| ☐ | ☒ | A description of all covariates tested |
| ☐ | ☒ | A description of any assumptions or corrections, such as tests of normality and adjustment for multiple comparisons |
| ☐ | ☒ | A full description of the statistical parameters including central tendency (e.g. means) or other basic estimates (e.g. regression coefficient) AND variation (e.g. standard deviation) or associated estimates of uncertainty (e.g. confidence intervals) |
| ☐ | ☒ | For null hypothesis testing, the test statistic (e.g. *F*, *t*, *r*) with confidence intervals, effect sizes, degrees of freedom and *P* value noted<br>*Give P values as exact values whenever suitable.* |
| ☒ | ☐ | For Bayesian analysis, information on the choice of priors and Markov chain Monte Carlo settings |
| ☒ | ☐ | For hierarchical and complex designs, identification of the appropriate level for tests and full reporting of outcomes |
| ☐ | ☒ | Estimates of effect sizes (e.g. Cohen's *d*, Pearson's *r*), indicating how they were calculated |

*Our web collection on statistics for biologists contains articles on many of the points above.*

## Software and code

Policy information about availability of computer code

**Data collection**

Commercial code was retrieved from online packages including DepMap (www.depmap.org), release noted in the manuscript, as well as Stringdb, PANTHER and Metascape.
X-Ray diffraction data were collected at -180C in house (CuKa X-Rays generated by a Rigaku Micro-Max 007 HF-X-ray generator) and at the Synchrotron beamlines 22-ID/NM (SER-CAT) and 23-ID (GM/CA) of the Advanced Photon Source, Argonne National Laboratory.

**Data analysis**

X-ray diffraction data: Data were reduced and scaled with XDS66 or DIALS67 and Aimless.68. The structures were solved by molecular replacement, structural refinement was carried out with PHENIX69 and model building with Coot70. Initial models for the small molecule ligands were generated with ligand restrains from eLBOW of the PHENIX suite. Figures were prepared using PyMOL. The coordinate sets and structure factors were deposited in the PDB (supplementary Table 5).

Dependency-STRING database Analysis:
Dependency analysis was performed using the 22Q4 release of the DepMap dataset, comprising seven medulloblastoma cell lines: UW228, DAOY, ONS76, D458, D425, D283MED, D341MED. Dependency was defined as a Chronos gene effect score of <-0.5, and gene lists were filtered for dependency in >2/7 medulloblastoma cell lines. Interaction networks were determined using the String database54, using medium confidence settings, network edges = confidence, no interactors, and hiding disconnected nodes (for JQ1, CCS1477) or showing disconnected nodes (A485). PANTHER was used to define gene annotations53, with unknown annotations assigned using individual assessment using the Genecards.org resource.

RNA-seq, String Analyses:
Sequences were aligned using HISAT2 (version 2.1.0)78 in paired-end mode with default parameters to build version hg19 of the human

genome to which the sequences of the ERCC synthetic spike-in RNAs (http://tools.invitrogen.com/downloads/ERCC92.fa) had been added. Per-gene expression was quantified using htseq-count79 with parameters "-i gene_id --stranded=reverse -f bam -m," and version 87 of the canonical GRCh37 gene list from RefSeq to which ERCC coordinates were added. TPM-normalized (Transcripts Per Million) expression was then computed for each gene and synthetic ERCC spike-in RNA. Per-gene exon length was calculated across all exons of all isoforms of each gene. The standard TPM-normalization strategy is: normterm = sum of (read count * read length / exon length) across all genes. TPM = read count * read length / exon length * 1e6 / normterm. We used a loess regression to normalize the TPM values across all samples of the batch, using only the spike-in values to fit the loess by the "affy" R package80 with function loess.normalize. This function allowed us to perform loess regression on a matrix of TPM values and used the ERCC subset of data for fitting the loess. The output from this was a matrix of normalized TPM values controlled by ERCC spike-ins.

ERCC-normalized expression of each gene after 6 hours of CCS1477, A485 or JQ1 was compared against its expression in DMSO-treated samples. Per-gene fold changes were calculated and statistical differential expression analysis seeking significance values for each gene was performed using DESeq2 with default parameters on raw counts of treated versus DMSO conditions, or comparing A485 and CCS1477-treated samples, using the ERCC probe read counts as controlGenes81. Genes were deemed significantly differentially expressed based on an adjusted p-value of <0.05 from DESeq2 analysis. Genes that were significantly differentially expressed in any treatment or cell line were selected for the k-means cluster heatmap in Fig 4a (DESeq2 adjusted $p < 0.05$, n = 5092). We used the elbow method82 to determine the k value in k-means cluster analysis.

Significantly differentially expressed genes (DESeq2 adjusted $p < 0.05$) in both cell lines were integrated to identify the union of high-confidence up or downregulated genes for each treatment subset. These genes served as input for analysis in METASCAPE83. Subsetted gene lists were used for analysis by GSEA using the Gene Ontology Hallmarks collection in MSigDB84, PANTHER53 or the String database54.

For manuscripts utilizing custom algorithms or software that are central to the research but not yet described in published literature, software must be made available to editors and reviewers. We strongly encourage code deposition in a community repository (e.g. GitHub). See the Nature Portfolio guidelines for submitting code & software for further information.

## Data

Policy information about availability of data

All manuscripts must include a data availability statement. This statement should provide the following information, where applicable:

- Accession codes, unique identifiers, or web links for publicly available datasets
- A description of any restrictions on data availability
- For clinical datasets or third party data, please ensure that the statement adheres to our policy

Data Availability: The RNAseq data generated in this study have been deposited in the Gene Expression Omnibus (GEO) database under SuperSeries accession number GSE233609 [https://www.ncbi.nlm.nih.gov/geo/query/acc.cgi?acc=GSE233609]. The x-ray crystallography data generated in this study have been deposited in the Protein Databank (PDB) database under PDB codes 8FV2 [https://doi.org/10.2210/pdb8FV2/pdb], 8FVF [https://doi.org/10.2210/pdb8FVF/pdb], 8FVK [https://doi.org/10.2210/pdb8FVK/pdb], 8FVS [https://doi.org/10.2210/pdb8FVS/pdb], 8FXA [https://doi.org/10.2210/pdb8FXA/pdb], 8FXE [https://doi.org/10.2210/pdb8FXE/pdb], 8FXO [https://doi.org/10.2210/pdb8FXO/pdb], 8GA2 [https://doi.org/10.2210/pdb8GA2/pdb] Source data are provided as a source data file.

## Research involving human participants, their data, or biological material

Policy information about studies with human participants or human data. See also policy information about sex, gender (identity/presentation), and sexual orientation and race, ethnicity and racism.

| | |
|---|---|
| Reporting on sex and gender | N/A |
| Reporting on race, ethnicity, or other socially relevant groupings | N/A |
| Population characteristics | N/A |
| Recruitment | N/A |
| Ethics oversight | N/A |

Note that full information on the approval of the study protocol must also be provided in the manuscript.

# Field-specific reporting

Please select the one below that is the best fit for your research. If you are not sure, read the appropriate sections before making your selection.

☒ Life sciences ☐ Behavioural & social sciences ☐ Ecological, evolutionary & environmental sciences

For a reference copy of the document with all sections, see nature.com/documents/nr-reporting-summary-flat.pdf

# Life sciences study design

All studies must disclose on these points even when the disclosure is negative.

| | |
|---|---|
| Sample size | Sample sizes were as standard for biological experiments with all experiments being performed at least with an independent biological n>3. For PRISM screening, experiments were performed by the PRISM screening facility at a single timepoint, with independent biological replicates n=3. |
| Data exclusions | For analyses of PRISM data, sample groups with fewer than 3 cell lines were excluded from lineage analysis. This was done in order to maximize reproducibility and avoid false conclusions related to low sample numbers. For analyses of cell-titer glo data, outlier datapoints were determined by the 1.5IQR method. |
| Replication | Experimental replicates were performed to n>=3 and all replicates indicated. |
| Randomization | Randomization is not appropriate for these biological experiments. |
| Blinding | Blinding was not performed. However, experiments where possible were reproduced by independent members of the study team blinded to each others results. |

# Reporting for specific materials, systems and methods

We require information from authors about some types of materials, experimental systems and methods used in many studies. Here, indicate whether each material, system or method listed is relevant to your study. If you are not sure if a list item applies to your research, read the appropriate section before selecting a response.

## Materials & experimental systems

| n/a | Involved in the study | |
|---|---|---|
| ☐ | ☒ | Antibodies |
| ☐ | ☒ | Eukaryotic cell lines |
| ☒ | ☐ | Palaeontology and archaeology |
| ☐ | ☒ | Animals and other organisms |
| ☒ | ☐ | Clinical data |
| ☒ | ☐ | Dual use research of concern |
| ☒ | ☐ | Plants |

## Methods

| n/a | Involved in the study | |
|---|---|---|
| ☒ | ☐ | ChIP-seq |
| ☒ | ☐ | Flow cytometry |
| ☒ | ☐ | MRI-based neuroimaging |

## Antibodies

| | |
|---|---|
| Antibodies used | Antibodies used in this manuscript include:<br>1. EP300 (Abcam) - catalogue #10485, used for western blotting at 1:1000.<br>2. BRD4 (Epicypher) - catalogue #13-2003, used for western blotting at 1:1000.<br>3. c-MYC (Cell Signaling Technology) - catalogue #5605, used for western blotting at 1:1000.<br>4. Beta-actin (Cell Signaling Technology) - catalogue #4967, used for western blotting at 1:1000.<br>5. Horseradish peroxidase–conjugated anti-rabbit (Santa Cruz Biotechnology) - catalogue sc-2357, used for western blotting at 1:5000.<br>6. Horseradish peroxidase–conjugated anti-mouse (Santa Cruz Biotechnology) - catalogue sc-542731, used for western blotting at 1:5000.<br>7. CBP (Cell Signaling Technology) – catalogue #7389, used for western blotting at 1:1000. |
| Validation | All antibodies were validated by the primary company selling as being specific for the noted target. All have previously been validated as specific using knockout, knockdown or overexpression assays in our own laboratories. |

## Eukaryotic cell lines

Policy information about cell lines and Sex and Gender in Research

| | |
|---|---|
| Cell line source(s) | Cell lines for PRISM screening were obtained from PRISM and are a part of the Cancer Cell Line Encyclopedia. Provenance is described in Yu C, et al. Nature Biotechnology, 2016. https://doi.org/10.1038/nbt.3460.<br>143B cells were obtained from ATCC and cultured in Eagle's MEM with 0.015mg/mL 5-bromo-2'-deoxyuridine and 10% FBS. Kelly cells were obtained from DSMZ and cultured in RPMI with 10% FBS. NCIH650 and NCIH2122 cells were obtained from ATCC and cultured in RPMI with 10% FBS. Kelly cells were obtained from DSMZ and cultured in RPMI with 10% FBS. RhJT and TE617T cells were a gift of the Broad Institute Pediatric Dependencies Project and cultured in RPMI with 10% FBS. HDMB03 and MB002 cells were generously provided by Till Milde (KiTZ, Heidelberg) and Yoon-Jae Cho (OHSU) and cultured in neurosphere medium |

| | |
|---|---|
| Authentication | All cells were validated to be free of mycoplasma spp. with routine testing for identity by short-tandem repeat testing. |
| Mycoplasma contamination | All cells were validated to be free of mycoplasma spp. with routine testing for identity by short-tandem repeat testing. |
| Commonly misidentified lines (See ICLAC register) | No commonly misidentified cell lines were used in this study. |

## Animals and other research organisms

Policy information about studies involving animals; ARRIVE guidelines recommended for reporting animal research, and Sex and Gender in Research

| | |
|---|---|
| Laboratory animals | Plasma pharmacokinetic (PK) profile of CCS1477 was evaluated in female CD-1 nude mice (Charles River) at approximately 8-12 weeks in age. All mice had consistent access to food and water and were housed at ambient temperature (20–25C) and humidity (40–60%) with 12-h light/12-h dark cycles. |
| Wild animals | No wild animals were used in this study. |
| Reporting on sex | PK profiles for CNS penetration of CCS1477 was performed only in female CD-1 nude mice. Sex as a variable for CNS penetration was not considered. |
| Field-collected samples | No field-collected samples were used in this study. |
| Ethics oversight | All animal studies were approved by the St. Jude Children's Research Hospital Animal Care and Use Committee and performed in accordance with best practices outlined by the NIH Office of Laboratory Animal Welfare. |

Note that full information on the approval of the study protocol must also be provided in the manuscript.

## Plants

| | |
|---|---|
| Seed stocks | N/A |
| Novel plant genotypes | N/A |
| Authentication | N/A |
