# Supplementary material for: Group 3 medulloblastoma transcriptional networks collapse under domain specific EP300/CBP inhibition
Source: Nat Commun. 2024 Apr 25;15:3483. doi: 10.1038/s41467-024-47102-0 (PMC11045757; doi:10.1038/s41467-024-47102-0)
Supplement: Supplementary file 4 — Source data [file 41467_2024_47102_MOESM4_ESM.zip › 433840_2_data_set_8777345_s325v5.zip!/Source Western Blots/Uncropped WB - Fig S3c.pdf]

Kelly cells - biotinylated CCS, CCSint pulldown #3 - 4-15-2022

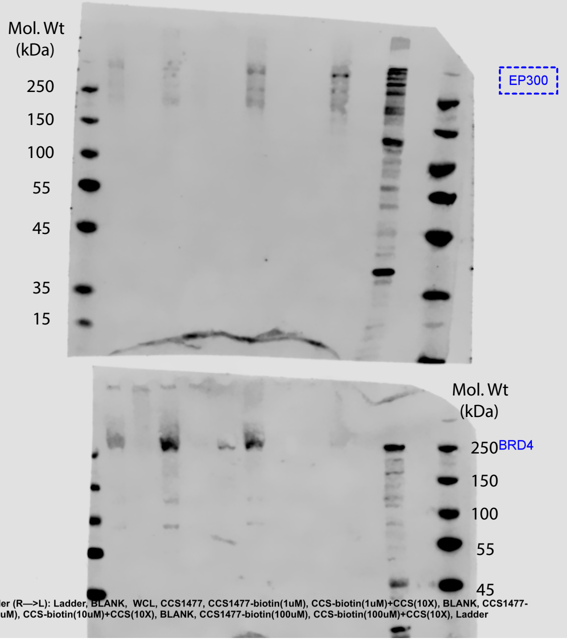

Lane order (R—>L): Ladder, BLANK, WCL, CCS1477, CCS1477-biotin(1uM), CCS-biotin(1uM)+CCS(10X), BLANK, CCS1477-biotin(10uM), CCS-biotin(10uM)+CCS(10X), BLANK, CCS1477-biotin(100uM), CCS-biotin(100uM)+CCS(10X), Ladder
