# Supplementary material for: Group 3 medulloblastoma transcriptional networks collapse under domain specific EP300/CBP inhibition
Source: Nat Commun. 2024 Apr 25;15:3483. doi: 10.1038/s41467-024-47102-0 (PMC11045757; doi:10.1038/s41467-024-47102-0)
Supplement: Supplementary file 4 — Source data [file 41467_2024_47102_MOESM4_ESM.zip › 433840_2_data_set_8777345_s325v5.zip!/Source Western Blots/Uncropped WB - Fig 4d.pdf]

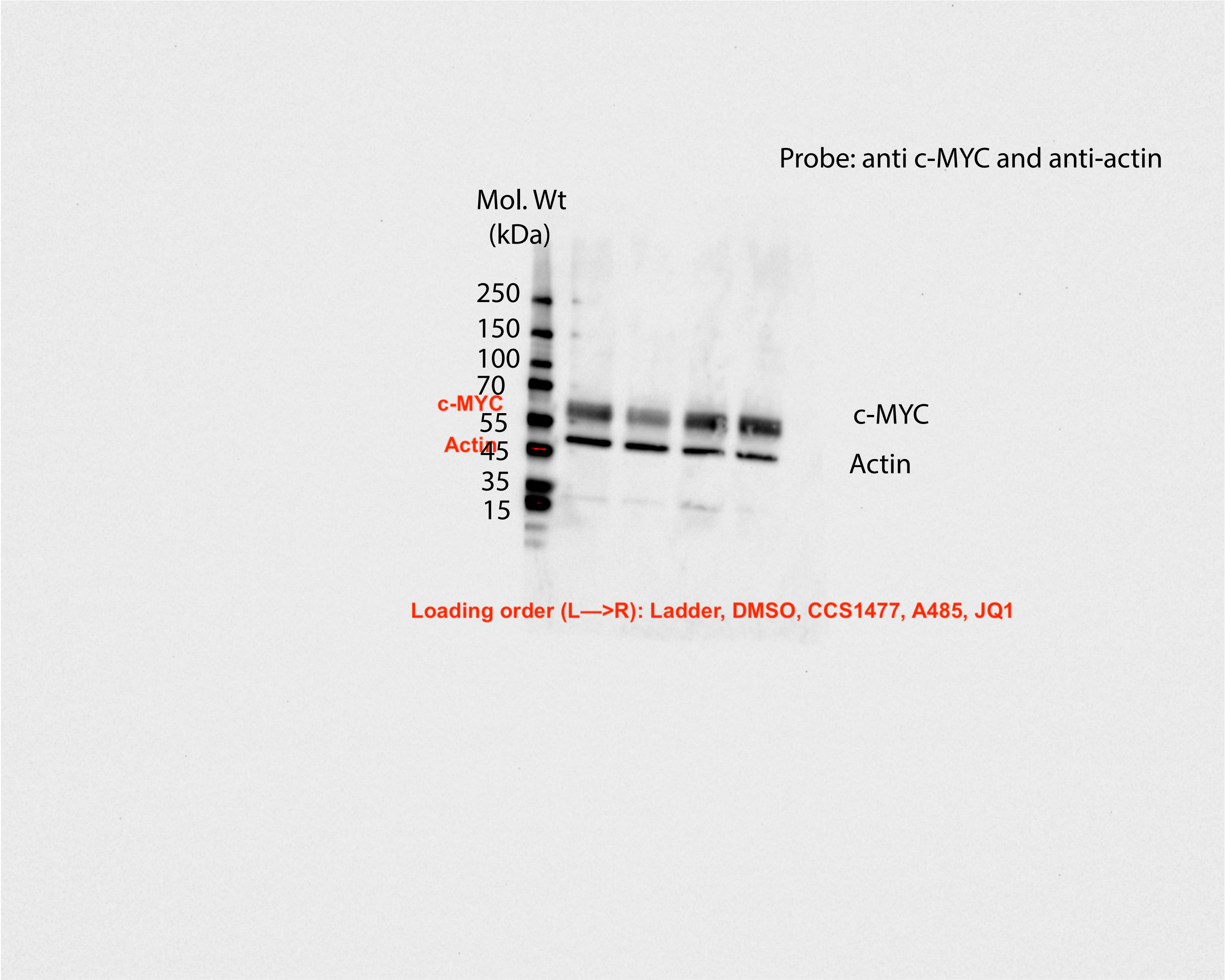
Probe: anti c-MYC and anti-actin
Mol. Wt
(kDa)
250
150
100
70
c-MYC
55
Actin
45
35
15
c-MYC
Actin
Loading order (L—>R): Ladder, DMSO, CCS1477, A485, JQ1
