# Supplementary material for: Group 3 medulloblastoma transcriptional networks collapse under domain specific EP300/CBP inhibition
Source: Nat Commun. 2024 Apr 25;15:3483. doi: 10.1038/s41467-024-47102-0 (PMC11045757; doi:10.1038/s41467-024-47102-0)
Supplement: Supplementary file 4 — Source data [file 41467_2024_47102_MOESM4_ESM.zip › 433840_2_data_set_8777345_s325v5.zip!/Source Western Blots/Uncropped WB - Fig 2g-EP300.pdf]

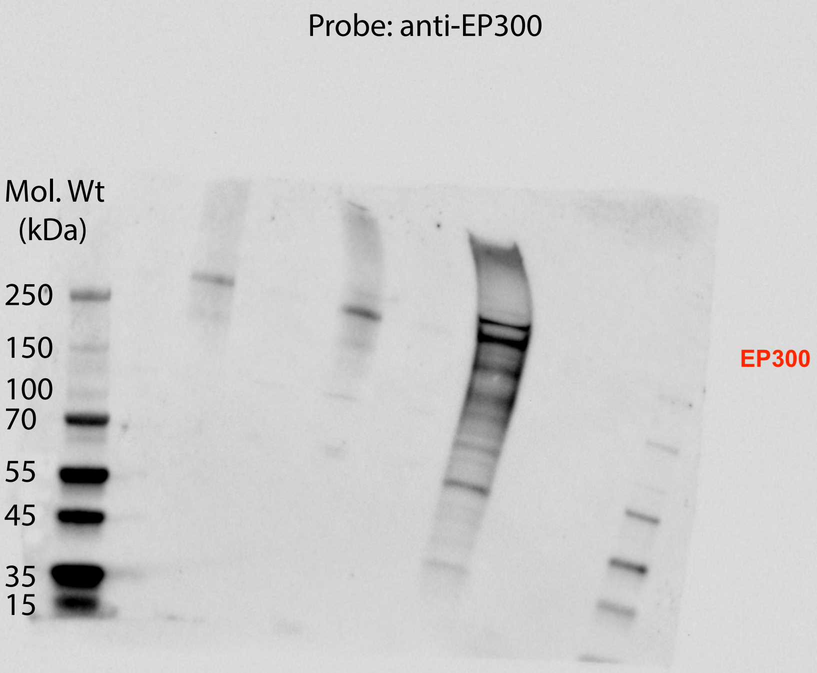

Loading Order: (R—>L): Ladder, Blank, Blank, Input, CCS1477-biotin (10uM) +CCS1477 100uM, CCS1477-biotin (10uM), CCS1477-biotin (1uM)+CCS1477 10uM, CCS1477-biotin (1uM), CCS1477 alone, Ladder
